# Rapid vertebrate speciation via isolation, bottlenecks, and drift

**DOI:** 10.1073/pnas.2320040121

**Published:** 2024-05-21

**Authors:** Andrew N. Black, Erangi J. Heenkenda, Samarth Mathur, Janna R. Willoughby, Brian L. Pierce, Sarah J. Turner, David Rizzuto, J. Andrew DeWoody

**Affiliations:** ^a^Department of Forestry and Natural Resources, Purdue University, West Lafayette, IN 47907; ^b^Western Association of Fish and Wildlife Agencies, Boise, ID 83719; ^c^Department of Evolution, Ecology and Organismal Biology, The Ohio State University, Columbus, OH 43210; ^d^College of Forestry, Wildlife, and Environment, Auburn University, Auburn, AL 36849; ^e^Natural Resources Institute, Texas A&M University, College Station, TX 77840; ^f^Department of Biological Sciences, Purdue University, West Lafayette, IN 47907

**Keywords:** pool-seq, whole-genome resequencing, demographics, conservation

## Abstract

Speciation is often driven by selective processes like those associated with viability, mate choice, or local adaptation, and “speciation genes” have been identified in many eukaryotic lineages. In contrast, neutral processes are rarely considered as the primary drivers of speciation, especially over short evolutionary timeframes. Here, we describe a rapid vertebrate speciation event driven primarily by genetic drift. The White Sands pupfish (*Cyprinodon tularosa*) is endemic to New Mexico’s Tularosa Basin where the species is currently managed as two Evolutionarily significant units (ESUs) and is of international conservation concern (Endangered). Whole-genome resequencing data from each ESU showed remarkably high and uniform levels of differentiation across the entire genome (global F_ST_ ≈ 0.40). Despite inhabiting ecologically dissimilar springs and streams, our whole-genome analysis revealed no discrete islands of divergence indicative of strong selection, even when we focused on an array of candidate genes. Demographic modeling of the joint allele frequency spectrum indicates the two ESUs split only ~4 to 5 kya and that both ESUs have undergone major bottlenecks within the last 2.5 millennia. Our results indicate the genome-wide disparities between the two ESUs are not driven by divergent selection but by neutral drift due to small population sizes, geographic isolation, and repeated bottlenecks. While rapid speciation is often driven by natural or sexual selection, here we show that isolation and drift have led to speciation within a few thousand generations. We discuss these evolutionary insights in light of the conservation management challenges they pose.

## Significance

We studied desert fish populations that nominally comprise a single species. High levels of genome-wide differentiation were observed, and coalescent-based species delimitation identifies two well-supported species. Combined with previously documented morphologic and ecological differences, our results indicate that these populations should be reclassified as distinct species that have diverged primarily due to neutral drift. This “drift speciation” event has occurred in the very recent evolutionary history of the lineage (~5 kya). The stochastic neutral processes associated with repeated bottlenecks have provided little opportunity for ecological speciation through local adaptation. The finding of two species emphasizes the conservation need to establish a refuge population and to maintain gene flow between natural and replicate populations of each species.

Evolutionarily significant units (ESUs) encompass lineages of genetically and ecologically distinct organisms that are effectively managed as intraspecific taxonomic units which merit increased conservation focus (1–4). Defining such conservation units is fraught with both political and biological challenges, but genomic data can refine these boundaries in natural populations (5–7). Population genomics can clarify the phylogeography and the timing of boundary emergence. Both are important as the cessation of gene flow ultimately puts ESUs or incipient species on different evolutionary trajectories, each subject to the vagaries of drift and selection (8). Rapid sympatric speciation is well-known (e.g., due to polyploidy or chromosomal rearrangements) (9), but allopatric speciation usually “arises gradually and incidentally as a result of mutation, genetic drift, and the indirect effects of natural selection driving local adaptation” (10). This paper is concerned with rapid evolution in allopatry, the relative roles of drift and selection in driving speciation, and the conservation management implications of our population genomic insights.

**Fig. 1.**
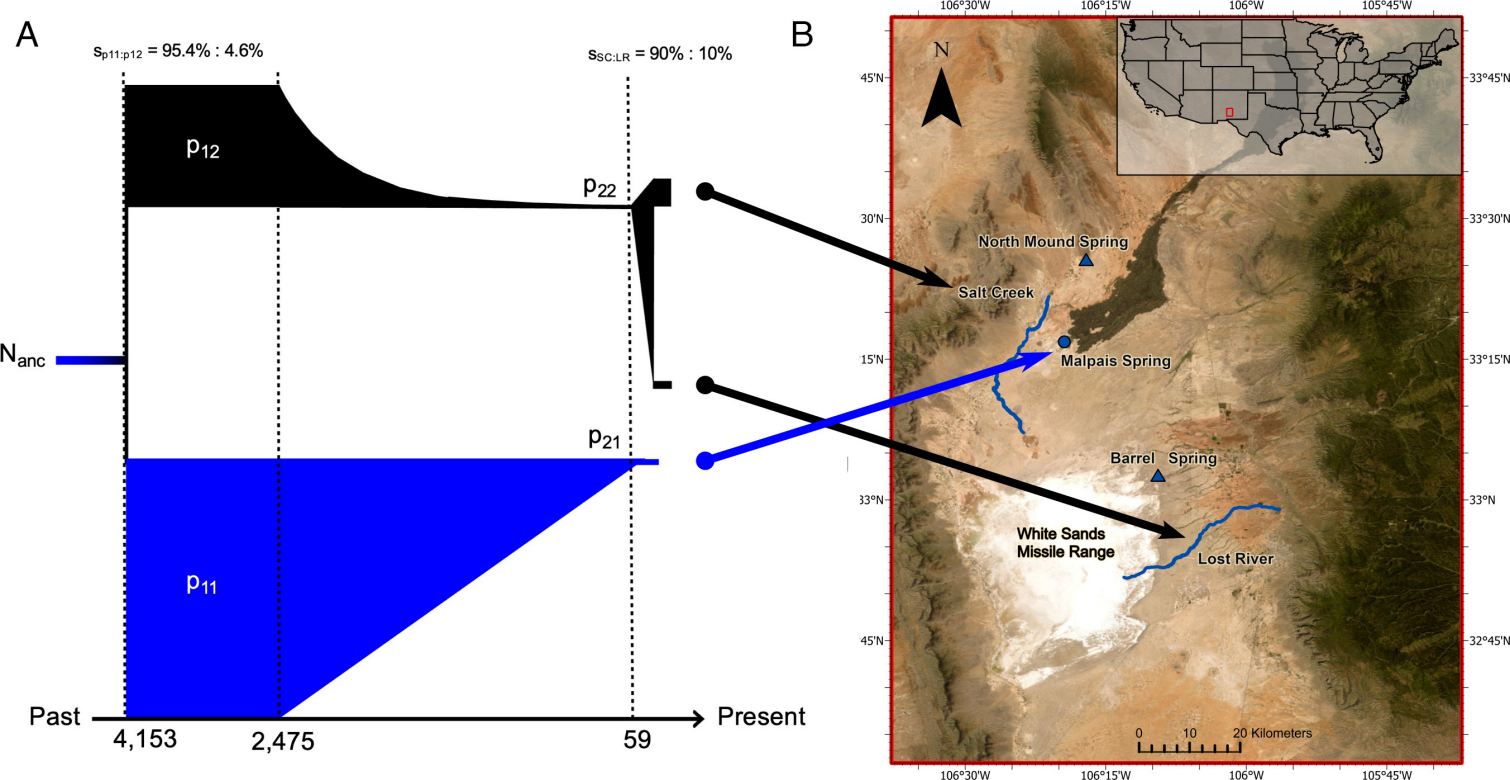
Demographic inference and sampling locations. (*A*) The most likely three-population demographic model indicates the ancestral population (*N_anc_*) split in a 95%:5% ratio around 4,153 B.P. to form incipient populations in MS, p_11_, p_21_ and SC, p_12_, p_22_, respectively. Both populations remained demographically stable for nearly 1,700 y and subsequently underwent strong bottlenecks 2,475 B.P. with the contemporary MS population being formed after a linear decline in the size of p_11_(p_21_). The SC and LR contemporary populations originated from the p_12_ population after undergoing an exponential decline in population sizes (p_22_); LR was established ~60 B.P. from ~10% of the p_22_ ancestors and contemporary SC pupfish contain ~90% of the p_22_ ancestry. Colors correspond to the two ESUs, with MS ESU1 = Blue and SC/LR ESU2 = Black. Population size estimates and 95% CI for each estimate are provided in *SI Appendix*, Table S1. These results show that a) pupfish in MS have been isolated for the last ~4,000 y and b) LR was recently established from the SC population, consistent with the known introduction history (12). (*B*) Map illustrating the geographic location of the three sampled *C. tularosa* populations. Two proposed relocation sites (18), North Mound Spring and Barrell Spring, were not sampled by us but are also included and symbolized by triangles.

The endangered (11) White Sands pupfish (*C. tularosa*) is a small desert killifish that is endemic to the Tularosa Basin in New Mexico where it inhabits four sites. Two of the four sites, Salt Creek (SC) and Malpais Spring (MS), are occupied by natural populations (Fig. 1). The other two sites, Lost River (LR) and Mound Spring, are anthropogenic in origin. The LR population was founded in 1970, likely by fish translocated from SC (12). The source of *C. tularosa* in Mound Spring is less certain, but allozyme and microsatellite marker data suggest that it was also founded from the SC population (13) between 1967 and 1973 (12). Two ESUs are currently recognized due in part to pronounced microsatellite differentiation (mean F_ST_ = 0.39 across two loci) as well as private alleles (13). Attempts to identify reciprocal monophyly of gene genealogies, a condition often required for ESU designation (2), have proven futile as single gene surveys have revealed little to no variation in mitochondrial control region DNA sequences (13). However, there is additional genetic support for the two ESUs in the form of significant heritable differences in body shape between fish at these sites (14, 15). There are substantial differences in water chemistry, hydrologic characteristics, and water flow between the MS (ESU1) and SC+LR (ESU2) sites (12, 16). This is important because adaptive variation is a critical component in shaping population discreteness and, as a result, ESU classification (4, 17).

Here, we used population genomic data from MS, SC, and LR pupfish to identify the primary evolutionary mechanism driving allopatric divergence between the two ESUs. We did so while simultaneously considering the effects of putatively neutral genomic regions and adaptive regions (19). We reconstructed the demographic history of each ESU, examined patterns of divergence and lineage sorting between ESUs, tested for signatures of selection (including at candidate genes that we expected to underlie local adaptation), and performed formal tests for species delimitation. Our genomic data indicate that isolation and drift, rather than selection for locally adapted variants, have been the primary drivers for substantial and rapid population divergence, leading to the evolution of incipient vertebrate species in only a few thousand generations.

## Divergence Times.

Previous modeling based on microsatellite data estimated that divergence of the two ESUs occurred 3,250 to 11,000 B.P. due to the vicariant Carrizozo Lava flow event that occurred 5,200 ± 700 B.P. (20, 21). To hindcast population demographies using whole-genome resequencing (WGR) data, divergence times and long-term effective population size (N_e_) were estimated from a three-population model constructed by simulating the joint site frequency spectrum (SFS) of the three sampled populations that comprise the two ESUs (ESU1 from MS; ESU2 from SC+LR). The most likely demographic model indicates a progenitor population size of *N_anc_* = 3,911 (95% CI = 3,644–4,178) and that this progenitor population split into two (p_11_ and p_12_) ~4,153 B.P. (3,871 to 4,436). Population p_11_ (the forebearers of MS) retained 95% of the ancestral gene pool, whereas population p_12_ (the forebearers of SC+LR) retained only ~5% of the ancestral variation (Fig. 1*A* and *SI Appendix*, Table S1).

Our SFS simulations (Fig. 1*A*) indicate that the ancestral MS population remained large and stable over time (N_p11_ = 64,866 [61,893–67,839]) whereas the ancestral SC/LR population was stable but only a quarter the size (N_p12_ = 17,070 [14,891–19,249]). Both ESUs underwent bottlenecks ~2,475 y ([2,263–2,687]) ago with p_11_ (MS, ESU1) declining linearly whereas p_12_ (SC+LR, ESU2) experienced strong exponential bottlenecks (Fig. 1*A*). The model reveals that the contemporary MS samples represent a population formed from the bottlenecked p_21_ population with substantially smaller effective sizes (N_MS_ = 182 [166–198]). In contrast, both the contemporary SC and LR populations (ESU2) were formed from the exponentially bottlenecked p_22_ ancestral population. Our analyses estimate the establishment of LR at 59 B.P. (95% CI = 48–70) from only 10% of the bottlenecked ancestral (p_22_) gene pool. Forebearers of fish from the contemporary SC site had the largest population sizes (N_SC_ = 8,714 [8,196–9,232]) that represents ~85% of the p_22_ population. A small sample of ~30 SC fish was used to establish the LR population in 1970 (i.e., 48 y before our 2018 LR sample collections), and this documented translocation event (12) coincides closely to the contemporary divergence time estimated by our demographic model (59 y; Fig. 1*A*).

The demographic history of these pupfish was undoubtedly shaped by geographic isolation due to the Carrizozo lava flow (21). The geologic history, our demographic model, and a Bayesian analyses (see below and *SI Appendix*, Table S2) are largely concordant and indicate that the two ESUs have been on independent trajectories for only ~1,000 to 5,500 y (also see *SI Appendix*, Table S2). Thus, all observed genomic differentiation between the two ESUs must have arisen in the last few thousand years.

### Geographic Variation and Genomic Divergence.

**Fig. 2.**
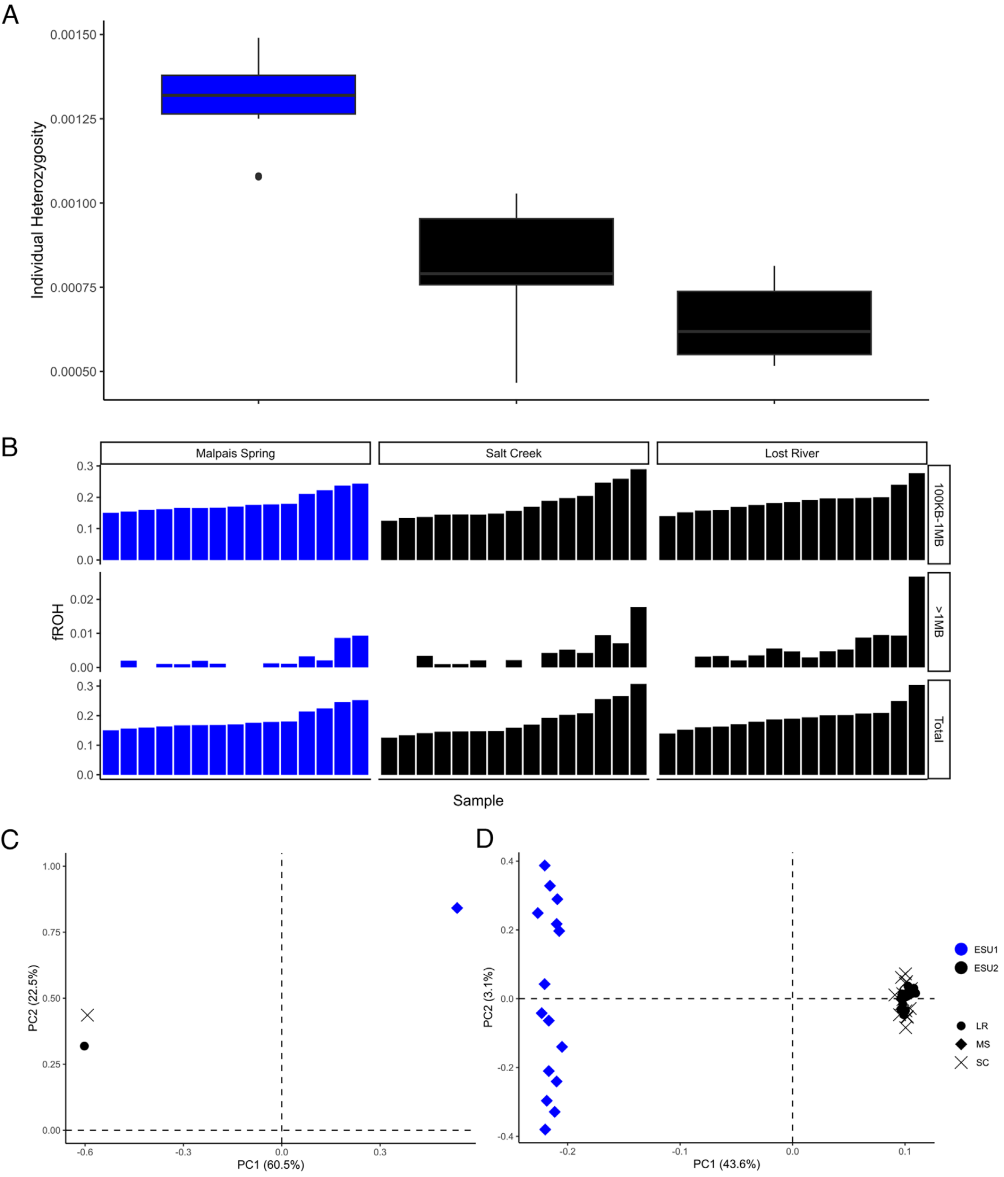
Genomic variation, autozygosity, and principal component analyses. (*A*) Boxplot (median, first and third quartiles) of individual heterozygosity estimates from 45 WGR samples, grouped by source population. (*B*) The proportion of the genome with Runs Of Homozygosity (fROH) for each of the 45 WGR samples, binned by length of the ROHs (100 kb to 1 Mb; >1 Mb; Total). (*C*) Principal component analysis of allele frequencies (maf > 0.05) derived from pool-seq and (*D*) WGR data with variation explained labeled on each axis for each sequencing approach. For plots *A*–*D*, colors represent the two historic ESUs and shapes represent the source population for individuals collected from SC, LR, and MS.

We quantified the distribution of genome-wide variation as the number of heterozygous sites/total number of sites for each fish, summarized by population (Fig. 2*A*). Consistent with Black et al. (22), individual heterozygosity among all 45 samples was very low and varied by a factor of three across sites, ranging from 0.00046 (LR) to 0.00149 (MS). Significant genome-wide differences in heterozygosity were observed across pairwise population comparisons (Wilcoxon signed rank test, W = 0 to 42, *P*-values < 0.003). Pupfish collected from MS (ESU1) exhibited higher levels of heterozygosity than observed in samples collected from SC or LR (ESU2; Fig. 2*A*). The artificially translocated population (LR) showed the lowest levels of heterozygosity, which was significantly reduced relative to its source population (SC; Fig. 2*A*).

ROH and cumulative ROH lengths among individuals indicate moderate levels of inbreeding (fROH = 0.125–0.307) among the three sites, with most ROHs identified as ancestral (ROH lengths = 100 kb to 1 Mb; Fig. 2*B*). There were no differences in fROH_100 kb–1 Mb_ between ESUs (Wilcoxon signed rank test, W = 213, *P*-value = 0.784) but the SC+LR/ESU2 gene pool harbors a higher proportion of long ROHs (fROH_>1 Mb_) compared to MS (Wilcoxon signed rank test, W = 313, *P*-value = 0.034). This is consistent with our demographic model and indicates slightly increased levels of contemporary inbreeding occurring within SC+LR (Fig. 2*B*). Considering all ROHs >100 kb across all populations (Fig. 2*B*), mean fROH was 0.187 (i.e., ~19% of the genome composed of homozygous blocks). This is only about one-third the ROH proportion seen in the critically endangered Devil’s Hole pupfish (fROH = 0.58) (23). To determine the potential impact of these ROHs, we evaluated genetic load by quantifying the accumulation of deleterious variants within fish from each site and found a high proportion of the high-impact variants were shared among all three sites, with no significant differences between impact classes among populations (*SI Appendix*, Fig. S4). This indicates that inbreeding has not been a strong agent of differential selection among these populations and that if present, inbreeding depression is likely to impact all three populations similarly.

A principal component analysis (PCA) was performed using allele frequencies across the genome. The PCA illustrates that MS samples cluster very distinctly from the SC and LR samples (Fig. 2 *C* and *D*). Principal component 1 clearly separated the two ESUs with a very high level of variance explained (Fig. 2*D*). We repeated the PCAs for the SC vs. MS comparison to assess the effects of selection versus drift (19) by partitioning allele frequencies among genic or nongenic regions. *SI Appendix*, Fig. S5 shows highly congruent patterns of sample clustering regardless of data partitions, indicating that genic regions are no more or less responsible for differentiation than nongenic (i.e., putatively neutral) regions of the genome.

To quantify the degree of differentiation among fish from different sampling sites, we calculated a pairwise fixation index (F_ST_, ranging from 0 to 1). Pairwise comparisons between the native SC and the translocated LR population (both ESU2) revealed moderate levels of genomic differentiation using pool-seq (F_ST_ = 0.046), WGR (F_ST_ = 0.142), and mitochondrial (F_ST_ = 0.198) sequences. Considering that LR was founded by SC in 1970 (12), the observed level of nuclear genomic differentiation (F_ST_ = 0.046–0.142) is high given that it manifested in only ~50 generations. A similar pattern has been observed between captive and wild populations of the Endangered Leon Springs pupfish (*C. bovinus*), which also exhibited moderate levels of differentiation (F_ST_ = 0.062) after a founding event and 15 y of separation (24). We conclude that this rapid differentiation is driven by genetic drift due to founder effects, variance in reproductive success, and demographic instability associated with desert ecosystems.

**Fig. 3.**
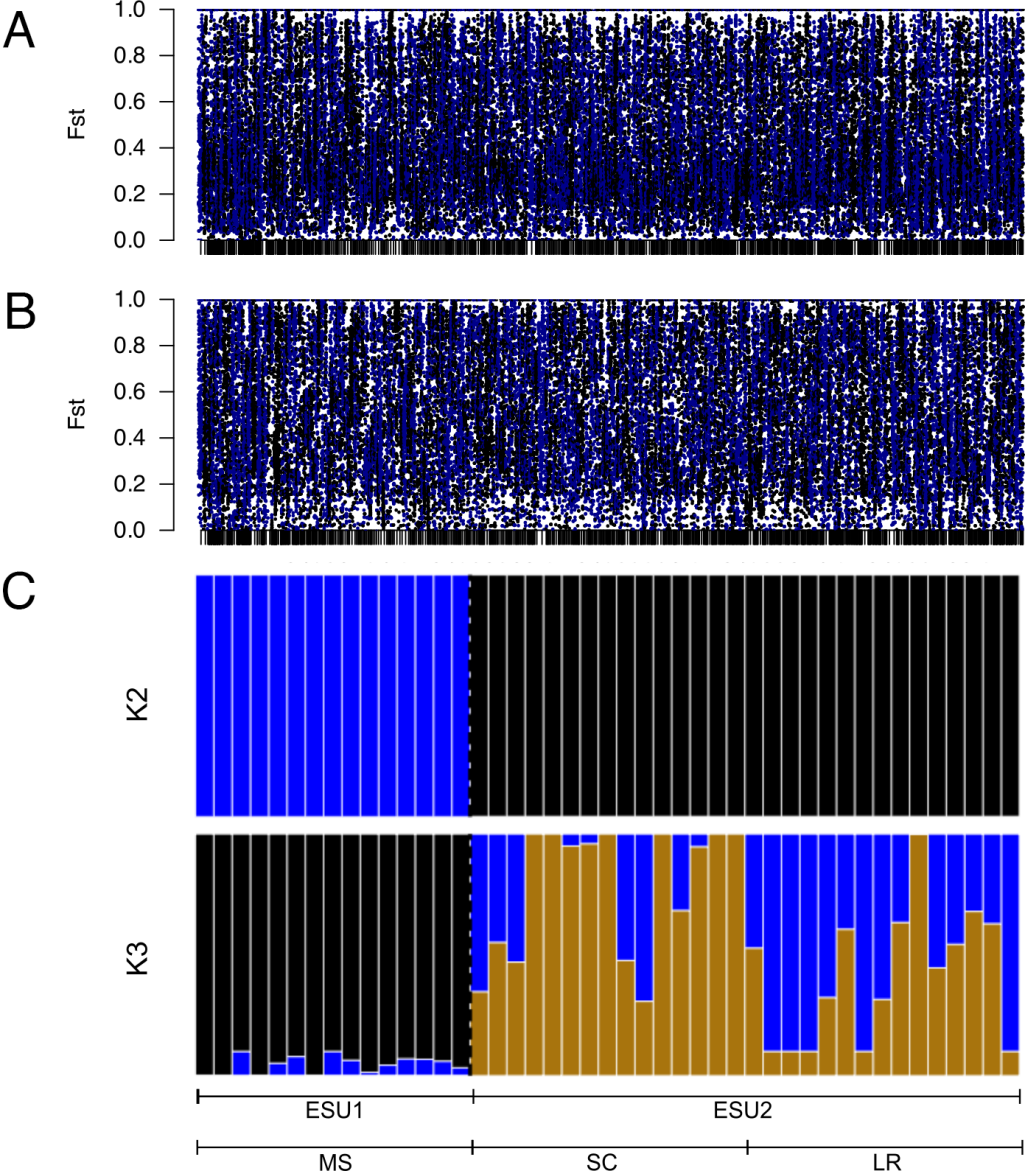
Genomic differentiation and population structure. Genome-wide differentiation (F_ST_) between SC and MS for pool-seq (*A*) and whole-genome resequencing (*B*) datasets. Results across 245 contigs (black vertical bars, with alternating blue and black colors) clearly demonstrate uniformly high levels of genome-wide divergence. (*C*) Individual admixture memberships (K = 2 to 3) of individuals from each ESU, underscored by the source population (LR = Lost River; SC = Salt Creek; MS = Malpais Spring).

Genomic differentiation between ESUs was nearly fourfold greater than F_ST_ values between populations from the same ESU. Specifically, the comparisons between ESU1 vs. ESU2 showed high levels of differentiation for both pool-seq (SC vs. MS; F_ST_ = 0.282 and LR vs. MS; F_ST_ = 0.275) and WGR datasets (SC vs. MS F_ST_ = 0.406 and LR vs. MS F_ST_ = 0.400, *SI Appendix*, Table S3). Mitochondrial genomes also exhibit remarkably high F_ST_ values between ESUs (SC vs. MS F_ST_ = 0.896 and LR vs. MS F_ST_ = 0.921), which reflects the nearly fixed differences in haplotype frequencies. Restricting analyses to the two ESUs (SC vs. MS) and evaluating sites contained in genic (F_ST_ = 0.300, 0.423) or nongenic (F_ST_ = 0.283, 0.410) regions revealed similar and elevated levels of differentiation for both pool-seq and WGR datasets, respectively. The high background levels of genome-wide differentiation between ESUs (Fig. 3 *A* and *B*) and similar levels of differentiation between genic and nongenic regions indicate that genomic divergence between ESUs is not limited to localized sections of the genome (e.g., structural arrangements) and that the differences in allele frequency spectra encompass most of the functional aspects within the genome (e.g., protein-coding genes).

Population structure was further evaluated by examining individual admixture proportions among all three sampling sites in an a priori-free manner, which assumes a model of *K* unknown clusters which represent genetic populations. As the Δ*K* method generally detects the highest level of population structure (25), two different approaches were utilized to examine nonhierarchical population clustering. Estimates of individual admixture proportions from a PCA (26) denoted two clusters, whereas the Δ*K* method identified three optimal clusters (27). Visual inspection of K = 2 indicated that individuals clearly grouped according to ESU designation, whereas K = 3 revealed substructure occurring within ESU2 (i.e., SC+LR; Fig. 3*C*). These analyses demonstrate that not only was divergence detected between ESUs (K = 2) but the differentiation that has arisen over the last ~50 y between source and replicate population were detected as well (K = 3), consistent with the F_ST_ results.

### Phylogenomics and Species Delimitation.

**Fig. 4.**
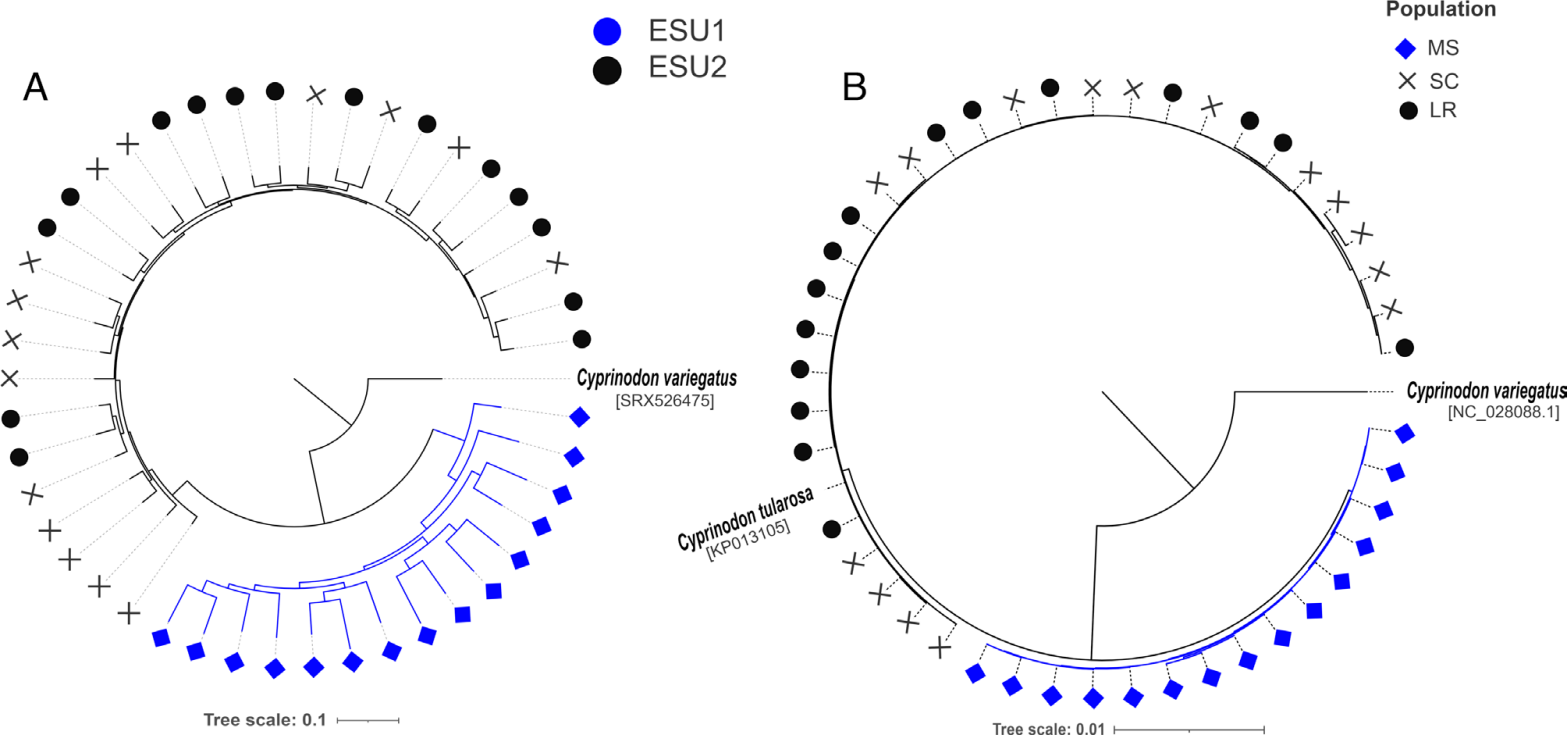
Maximum-likelihood trees derived from nuclear and mitochondrial genomes. (*A*) Maximum likelihood nuclear tree from whole-genome resequencing of 45 pupfish from this study and *C. variegatus* reads from NCBI. (*B*) Maximum-likelihood tree derived from aligned mitogenomes of 45 pupfish used in this study and *C. tularosa/C. variegatus* mitogenomes from NCBI.

Neutral gene genealogies are expected to transition from polyphyly to paraphyly to reciprocal monophyly. This natural progression of lineage sorting helps define both ESUs and species (2, 4, 28). We generated maximum-likelihood trees of n = 45 nuclear and mitochondrial genomes and found that the two ESUs are reciprocally monophyletic (Fig. 4 and *SI Appendix*, Fig. S6).

As expected, our population genomic results strongly support the original demarcation that identified two ESUs (13). However, because of the recent geographic and demographic isolation of these sites (21) (Fig. 1), we did not anticipate the remarkable genomic differentiation we found between the two ESUs. This differentiation is reflected in the high F_ST_ values estimated with nuclear (F_ST_ ≈ 0.40) and mitochondrial (F_ST_ ≈ 0.90) sequences, the high levels of variance explained by the principal component analysis (PC1 = 43.6% between ESUs for WGR data), the individual ancestry coefficients, and reciprocal monophyly of whole-genome trees (both nuclear and mitochondrial).

This cryptic genomic differentiation observed between the nominal *C. tularosa* ESUs is buttressed by the morphological differences among fish from the two ESUs (14, 15) and the myriad environmental differences between ESU sites (12, 16). Thus, we shifted from an intraspecific assessment of genomic diversity to an interspecific evaluation by conducting formal tests of species delimitation. We used the Bayesian Phylogenetics and Phylogeography (29, 30) approach to delimiting species using two separate datasets. One consisted of 1,000 nuclear DNA (nDNA) loci and the other of the full mitogenome (mtDNA). Two different species delimitation algorithms (rj1, rj0) were run using a guide tree (A10) and the nDNA loci by estimating species trees (A11). All nDNA analyses supported the presence of two distinct species (MS vs. SC/LR) with a posterior probability (pp) of 1. Analysis of the mtDNA genomes revealed similar probabilities (pp = 0.87 to 1). To test the robustness of these results, we conducted serial A11 analyses of five distinct subset sets of nDNA loci and again detected two species (pp = 0.83 to 1). In summary, all formal delimitation analyses consistently identified the same two clusters of species which corresponded to established ESUs, regardless of the datasets (1,000/20 nDNA, mtDNA), the analyses (A11, A10), or the delimitation algorithm (rj1, rj0) employed (*SI Appendix*, Table S4).

### Genomic Signatures of Local Adaptation.

Genomic signals of local adaptation could exist given the significant evolutionary time that is typically associated with the evolution of reciprocal monophyly (31). Probabilities of reciprocal monophyly increase over time and are proportional to N_e_; it takes an average of 4N_e_ generations for neutral, haploid, asexual markers like mitochondrial genomes to stochastically achieve reciprocal monophyly (28, 32) and longer for diploid, recombining, and adaptive autosomal markers. Consistent with the demographic model (Fig. 1*A*), the fact that *C. tularosa* exhibits complete lineage sorting in both nuclear and mitochondrial genomes (Fig. 4) suggests that evolutionary (i.e., long-term)-effective population sizes have been small given the recent timescales involved (~4,000 y) since the cessation of gene flow between ESUs. This rapid divergence limits the temporal opportunity for selection, and the variable nature of desert fish population cycles (often driven by rainfall or lack thereof) further reduces the efficacy of selection when N_e_ is small. Kimura and Ohta (33) showed that selection is a potent evolutionary force only when N_e_(*s*) > 1, where *s* is the selection coefficient, and N_e_ tends to be exceptionally small when population sizes fluctuate widely because N_e_ is determined by the harmonic mean of census size over time (i.e., the reciprocal of the average of the reciprocal of census sizes).

Because of these theoretical limitations on the ability of selection to shape local adaptation, we conducted genome-wide tests for signatures of selection using a sliding-window outlier approach. Concerned that saturation of F_ST_ values might impede such outlier tests, we also employed a more conservative and targeted approach. Salinity varies substantially between ESUs; mean salinity at LR is 26.9 ppt, 15.9 ppt at SC, and 5.9 ppt at MS (13). Thus, we predicted that genes involved in osmoregulation should be candidate genes because salinity is known to be a strong selective force in many contexts (34, 35). We identified an a priori list of 34 candidate genes from a previous study on transcriptional dynamics of ion transporters and aquaporins in the gill of the desert Amargosa pupfish (*C. nevadensis amargosae*) during rapid salinity change (36). This list includes osmoregulatory genes, aquaporins, solute carriers, and ion cotransporters, as well as the phosphoguluconate dehydrogenase (*pgd*) gene (*SI Appendix*, Fig. S7) that encodes an allozyme locus previously examined in *C. tularosa* (*SI Appendix*, Table S5).

We conducted a nonoverlapping sliding window analysis (window size = 50 kb) of genome-wide F_ST_ between ESUs to identify genes potentially responsible for local adaptation. After quality filtering, we removed twelve windows (0.058% of the total) and subsequently found two candidate genes in the top 2.5% of F_ST_ windows (*Ca12* and *nkain2*; *SI Appendix*, Fig. S8*A*). Both appear to have functional relevance to salinity differences between ESUs so we estimated pairwise F_ST_ and the neutrality test statistic, Tajima’s *D* (37) in the two contigs containing these genes. We also calculated D_XY_ on a per‐SNP basis to investigate the patterns of absolute genetic divergence in these genic regions of interest. As absolute divergence is unaffected by diversity levels present in allopatric populations (38), D_XY_ is typically expected to be similar within and outside F_ST_ peaks. However, when polymorphisms are sorted unequally among populations, elevated D_XY_ and F_ST_ peaks can both occur. Furthermore, selective sweeps and background selection could result in reduced D_XY_ peaks but elevated F_ST_ peaks (38). D_XY_ was neither elevated nor reduced near *Ca12* or *nkain2* when compared to the genomic background (*SI Appendix*, Fig. S8*B*). A negative Tajima’s *D* was observed for *ca12* in ESU1 (MS), but no deviation from zero was detected in ESU2 (SC+LR). In the *nkain2* region, both ESUs exhibited deviations from neutrality, with ESU2 showing a negative deviation and ESU1 showing a combination of positive and negative deviations. However, we observed no clear pattern indicative of natural selection (*SI Appendix*, Fig. S8*B*).

### Drift Speciation and Management Considerations.

Despite the identification of two candidate genes that could underlie local adaptation, these pupfish genomes are generally depauperate of classic differentiation islands (Fig. 3 *A* and *B* and *SI Appendix*, Fig. S8*A*) (38). Instead, the pronounced differentiation between these ESUs has been driven primarily by recurring bottlenecks that lead to extreme and rapid genetic drift, effectively obscuring signatures of selection (39). Fluctuations in population sizes are common in many *Cyprinodon* species (23, 40). Thus, drift is expected to contribute heavily to genetic differentiation among geographic sampling sites (Fig. 1*B*). This pattern is apparent in our dataset by the lack of discrete islands of differentiation (Fig. 3 *A* and *B* and *SI Appendix*, Fig. S8*A*) and by rapid and complete lineage sorting of both nuclear and mitochondrial genomes (Fig. 4).

Hohenlohe et al. (41) studied freshwater populations of the three-spined stickleback (*Gasterosteus aculeatus*) that have been separated since the retreat of the last glacial maximum less than 20,000 B.P. Assuming a generation time of 2 y, this is roughly 10,000 generations and not dissimilar to the timescale of isolation between *C. tularosa* ESUs. However, the mean genome-wide nuclear F_ST_ among freshwater populations of three-spined stickleback was only 0.099 (41) compared to the ~0.40 we observed between *C. tularosa* ESUs. Despite being isolated for only half as many generations, differentiation is ~4× greater in *C. tularosa* (i.e., a ~8X rate of divergence difference between pupfish and stickleback). Furthermore, stickleback genomes displayed clear genomic signals of local adaptation that were not apparent in *C. tularosa*, highlighting the strength of drift relative to selection in the pupfish. Rapid ecological speciation is often associated with natural or sexual selection (see, e.g., refs. 42 and 43), but here we show that nonecological speciation (44) by isolation and drift can produce incipient species within a few thousand generations.

The rapid divergence between ESUs has conservation implications. If (after the initial founding event) there have been subsequent translocations of SC fish into LR (45), few if any of these fish appear to have survived and reproduced based on the observed F_ST_ values. Furthermore, there is significantly reduced genome-wide diversity in the recipient population (LR) compared to the source population (SC). Careful monitoring of introduced fish to evaluate postrelease mortality, survivorship, and breeding success is necessary if LR is to serve as a viable replicate population for SC. We note that all three populations had very low levels of genomic diversity, as evidenced by the identification of only ~200 to 400k genomic polymorphisms and overall heterozygosity levels ~0.009 (see also ref. 22). To our knowledge, there is no established safeguard against the extirpation of the MS population. As insurance, we recommend that managers consider the founding of a replicate (or captive) MS population because it contains most of the ancestral variation in any of the sampled populations. At least two uninhabited natural water sources, North Mound Spring and Barrel Spring, are potential candidate sites because they are similar to Malpais Spring in water chemistry (18). The establishment of refuge and captive populations have already been successfully implemented in other pupfish species such as the Devils Hole pupfish (23) and the Leon Spring pupfish (24) and could be implemented in this system as well.

Two distinct *C. tularosa* ESUs have long been recognized based on ecology, morphology, and marker-based genetic data (13, 14). Fish from these two ESUs also differ in meristic traits including scale, lacrimal pore, and gill raker counts (16). Our population genomic data provide even more resolution into the pronounced differences between these ESUs as evidenced by remarkably high and uniform levels of differentiation (i.e., F_ST_) across the genome, reciprocal monophyly in both nuclear and mitochondrial trees, and conclusive evidence for species delimitation. While mean absolute divergence between MS vs. SC was relatively low (0.127%), it is similar in magnitude to mean divergence between other recognized species of pupfish (*C. diabolis* vs. *C. nevadensis;* *SI Appendix*, Table S6).

The pupfish from the two ESUs considered in our study can hybridize and produce F1 offspring (15). However, we note that just as in many other taxa (46), interspecific hybridization and introgression are widespread among formally recognized pupfish species (47–50). Theoretical models and empirical data indicate that when speciation occurs, reproductive isolation is expected to evolve and to reinforce the incipient species boundaries (51–53). However, these ideas are often based in part (either explicitly or implicitly) on natural selection. For example, the evolution of reproductive isolating mechanisms [(e.g., postzygotic barriers due to Dobzhansky–Muller incompatibilities such as hybrid sterility or inviability, which are known to drive speciation) (54)] is driven by selection. Our data provide compelling evidence that biological populations may become sundered by vicariant events (e.g., lava flows) and that subsequent isolation and demographic stochasticity can rapidly produce daughter species whose genomes diverge nearly entirely by drift rather than selection.

The evolution of these two species has occurred in only a few thousand years, far more rapidly than typically observed in vertebrates (55, 56). The collective body of evidence clearly indicates these two pupfish ESUs qualify as distinct species using a wide variety of properties (e.g., monophyly, fixed qualitative differences, phenetic/quantitative differences, niche differences, etc.) and species concepts (57). Thus, we think they should be formally designated as two distinct species that each merits its own unique Latin binomial. We think that ESU1 should remain the White Sands pupfish (*C. tularosa*) because Malpais Spring is the type locality of the species (16), because it contains most of the ancestral genomic diversity and because of the tree topologies (Fig. 4). We also call for a (future) taxonomic revision that provides a formal species description of ESU2 and assigns it a new name. We respectfully suggest that ESU2 should be designated “the Enchanted pupfish” because they are endemic to New Mexico (where the state nickname is “the Land of Enchantment”), because of its crypsis, and because it serves to highlight the fascinating allure of drift speciation.

## Methods

Here, we only summarize the methods, but comprehensive details are provided in *SI Appendix*. Briefly, pupfish were collected from MS (n = 30), LR (n = 30), and SC (n = 30). Whole-genome library preparation and sequencing were conducted on individually barcoded samples (n = 15 from each population) and on three pools of samples (n = 30 from each population). Quality controlled reads were aligned to the *C. tularosa* reference genome (22) with bwa (58) and were processed using Poolparty (59) and Angsd (60), which were also used to estimate individual heterozygosity and pairwise differentiation. Population structure was evaluated using PCAngsd (61), NGSadmix (62), and runs of homozygosity were identified using bcftools (63). Nuclear and mitochondrial genomic trees were created using iqtree2 (64). Mitochondrial genomes were reconstructed using Mitobim (65). Species Delimitation was conducted using Bayesian Phylogenetics and Phylogeography (29, 30) and demographic history was modeled using gadma (66). Genetic load was evaluated using snpeff (67), and species divergence was estimated using NGStools (68).

## Supplementary Material

Appendix 01 (PDF)

## Data Availability

All pool-seq and WGR sequencing reads from this study are available from the National Center for Biotechnology Information Sequence Read Archive under BioProject PRJNA909848 (69). All scripts and commands used to analyze the data and generate figures are available on Github (https://github.com/Andrew-N-Black/WSP_popgen) (70).
