# Supplementary material for: Rapid vertebrate speciation via isolation, bottlenecks, and drift
Source: Proc Natl Acad Sci U S A. 2024 May 21;121(22):e2320040121. doi: 10.1073/pnas.2320040121 (PMC11145251; doi:10.1073/pnas.2320040121)
Supplement: Supplementary file 1 — Appendix 01 (PDF) [file pnas.2320040121.sapp.pdf]

Supporting Information for

Rapid vertebrate speciation via isolation, bottlenecks, and drift

Andrew N. Black[a,b†], Erangi J. Heenkenda[a†], Samarth Mathur[c], Janna R. Willoughby[d], Brian L. Pierce[e], Sarah J. Turner[e], David Rizzuto[e], J. Andrew DeWoody[a,f]

a. Department of Forestry and Natural Resources, Purdue University, West Lafayette, Indiana, USA
b. Western Association of Fish and Wildlife Agencies, PO Box 190150, Boise, ID, USA
c. Department of Evolution, Ecology and Organismal Biology, the Ohio State University, Columbus Ohio, USA
d. College of Forestry, Wildlife, and Environment, Auburn University, Auburn, Alabama, USA
e. Natural Resources Institute, Texas A&M University, College Station, Texas, USA
f. Department of Biological Sciences, Purdue University, West Lafayette, Indiana, USA
[†] These authors contributed equally to this work.

This PDF file includes:

- Supporting text
- Figures S1 to S8
- Tables S1 to S6
- SI References

Sample Collection

Non-lethal dorsal fin clips were obtained from n=90 mixed sex *C. tularosa* equally sampled among the three target populations: Malpais Spring (MS=30; collection date 5/2019), Lost River (LR=30; collection date 10/2018), and Salt Creek (SC=30; collection date 5/2017). Sampling methodology was approved by Texas A&M Animal Use Protocol AUD-2018-024A and followed rules outlined in the New Mexico Department of Game and Fish permit NMDGF Sci#3335.

Library Construction and Sequencing

Genomic DNA (gDNA) was extracted from the n=90 collected *C. tularosa* using a DNeasy blood and tissue kit (Qiagen) and quantified using fluorometry (Quibit; Thermo Fisher Scientific) prior to preparing three pooled genomic libraries (corresponding to the three focal populations (MS, LR, SC), each consisting of gDNA from n=30 individuals. These three pool-seq libraries were then prepared and sequenced at Purdue University's Genomics Core Facility using a S1 reagent kit on an Illumina NovaSeq 6000. To enable the calculation of genotype-based metrics, whole genome resequencing (WGR) data was also generated for n=45 (MS=15, SC=15, LR=15) of the n=90 gDNA extracts used for the three pool-seq libraries. That is, half of the pool-seq samples were also individually sequenced. WGR samples were processed at two different sequencing facilities. Fifteen samples (five from each population) were prepared using dual-indexed libraries with Illumina Nextera reagents (following the manufacturer's protocol) and sequenced using a S1 reagent kit across 2 lanes of an Illumina NovaSeq 6000 at Purdue University's Genomics Core Facility. The other thirty samples (10 from each population) were prepared using the Hyper Library construction kit from Kapa Biosystems (Roche) and sequenced using a S4 reagent kit on an Illumina NovaSeq 6000 at the University of Illinois (Roy J. Carver Biotechnology Centre).

Quality control

Prior to read filtering and mapping, the *C. tularosa* reference genome (1) was downloaded and parsed to reduce the negative impact of aligning reads to highly repetitive or ambiguous regions. Repetitive sequences were identified by extracting coordinates contained in the RefSeq Repeatmasker file (GCF_016077235.1_rm.out.gz). Regions were labeled as ambiguous if they had a mappability <1, as determined using 100-mers with GENMAP v.1.3.0 (2). Contigs<100kb in length were removed and a file containing non-repetitive unambiguous windows was created with BEDTOOLS (3) and used to filter binary alignment mapping files (see below). Quality assessment of both Pool-seq and WGR reads were conducted by visual examination of FASTQC (https://www.bioinformatics.babraham.ac.uk/projects/fastqc/) and MULTIQC (4) base score distributions and adapter content.

Pool-seq

The Pool-seq paired-end reads (2x151) were preprocessed using the *PPalign.sh* script from the POOLPARTY pipeline (5). Briefly, this bash script leverages BBDUCK (sourceforge.net/projects/bbmap/) to trim adapters and remove low quality bases, BWA v.0.7.17 (6) to align reads, SAMBLASTER v.0.1.26 (7) to remove duplicate reads, SAMTOOLS v.1.8 (8) to sort and filter alignments, and BCFTOOLS v.1.8 (9) to call variants. Parameters were set to a mapping quality of ≥20 (MAPQ=20), a SNP quality ≥20 (SNPQ=20), a base call accuracy ≥20 (BQAL=20), a minimum depth≥10 (MINDP=10), and an insertion/deletion window of 10 (INWIN=10) to remove potential false variants around indels. Furthermore, only alignments contained within the non-repetitive unambiguous regions were retained for downstream analysis (by using SAMTOOLS view -l bed file).

After removing identified adapters and low-quality bases, 176M mean (min-max=120M-217M) pool-seq reads remained, which all had a 100% alignment rate to the *C. tularosa* reference genome. Following mapping, binary alignment files were sorted based upon read coordinate and filtered for low base call accuracy, duplicate reads, low mapping quality and repetitive or ambiguous regions. The quality filtered pool-seq alignments had a mean depth of coverage of 13.8x (SC), 23.0x (LR), and 25.0x (MS) with a breadth of 90.4-90.8%. Pool-seq read alignment, variant calling, and quality filtering resulted in 396,648 variant sites with a minor allele frequency above 0.05 among the three populations.

Following alignment and filtering of the pool-seq data, the *.sync* file (which contains allele counts at each site for each population) and the *.fz* file (which contain allele frequencies at each site for each population) were used as input for the POOLPARTY script *PPanalyze.sh.* This script was used to leverage the program POPOOLATION2 (10) to: 1) quantitate population differentiation (F_{ST}) among a) all variants, b) genic, and non-genic sites, and 2) perform a principal component analysis using allele frequency data from the three pool-seq libraries. Parameterization included a minimum (MINCV=10) and maximum coverage of 100 (MAXCV=100), a minor allele frequency cutoff of 0.05 (MAF=0.05) and designated the number of individuals per pool (NIND=30).

Whole Genome Resequencing (WGR)

The WGR paired-end (2x151) reads were preprocessed using TRIMMOMATIC v.036 (11) by clipping identifiable Illumina adapters, removing low quality bases (Phred <20) found within the first (LEADING: 20; 5) or the last (TRAILING: 20; 3) 20-nucleotides, and discarding any processed read under 30-nucleotides in length (MINLEN:30). Fastq pair-aware quality trimming resulted in 80M mean WGR reads (min-max= 35M-109M) among the 45 samples. Quality trimmed WGR reads were then aligned to the *C. tularosa* RefSeq genome (GCF_016077235.1) using the BWA v.0.7.17 mem algorithm. Overall, samples

had a mean mapping rate of 99.4% (98.9%-100%) prior to any alignment filtering. Mapped reads were then processed using GATK v.3.6.0 (12), by sorting reads based upon genomic coordinate, marking duplicates and remapping reads around insertions/deletions to increase alignment accuracy (13). SAMTOOLS (v.1.8) bitflags (italicized) were used to remove unmapped (*4*), secondary (*256*), QC failed (*512*), duplicate (*1024*) and supplementary (*2048*) alignments from non-repetitive unambiguous regions specified in the bed file. After implementing these stringent alignment filters, samples had a 6.94x mean coverage rate for WGR individuals (min-max=3.34x-9.33x) and a mean breadth of 89.6% (min-max=86.1-90.4%).

Quality filtered WGR alignment files were then analyzed using ANGSD v.095 (14). To guide parameter selection and detect any inherent population level bias in the sequence data, phred score and coverage distributions were generated and plotted prior to processing (Figs. S1-S3). The SAMTOOLS variant calling method implemented in ANGSD was chosen to estimate Genotype Likelihoods (-GL 1). Bases with a phred score less than 35 or below (-setMinDepth 275) or above (-setMaxDepth 425) global depth thresholds were excluded. To reduce erroneous alignments from producing false variants around insertions and deletions, the base alignment quality algorithm (-baq 1) was utilized (9), mapping qualities of reads with excessive mismatches were adjusted (-c 50) and only properly paired reads with a mapping quality ≥30 were retained. To ensure sites were well represented among samples from all three populations, only those present in at least 80% of the samples (36 samples) with a minor allele frequency of 0.05 and a p-value cutoff of 10^{-6} were retained (but see genomic diversity section below). Overall, 215,846 sites were retained after WGR filtering.

Individual heterozygosity (*H)* was calculated from the WGR data using the site frequency spectrum (SFS). To minimize distortion of the SFS, minimal filtering was utilized for diversity metrics (-minQ 35). *H* is simply the number of heterozygous sites divided by the total number of sites for each sample (i.e., the proportion of heterozygote sites). Following tests for normality, significance between pairwise mean heterozygosity was assessed using a Wilcox signed rank test using *dplyr* v.1.09 and *ggpubr* v.0.40 in R. Results were visualized with *ggplot2* in R.

The Genotype Likelihoods produced by ANGSD were used to generate a covariation matrix as input for an individual principal component analysis with PCANGSD v.1.10 (15) and were plotted with *ggplot2* v.3.36 (16) in R v.3.42 (Team, 2019). To examine population structure among the three sampling sites, individual admixture proportions were estimated using NGSADMIX v.0.95 (17) for up to 7 populations and membership graphs were generated using the R package *pophelper* v.2.3.1 (18). Using the STRUCTURE model that minimizes deviations from Hardy-Weinberg equilibrium, the optimal number of genetic populations (K) was estimated from likelihood values using the ΔK method (19). The optimal K was also determined using a model-free approach based upon

principal component loadings using PCANGSD. Weighted pairwise F_{ST} of quality filtered sites was conducted by estimating the site allele likelihood (-dosaf 4) for each population (SC, LR, MS), followed by the folded 2D site frequency spectrums (realSFS) with ANGSD. Mean pairwise weighted F_{ST} was then calculated among sites located genome-wide as well as genic and non-genic regions.

Nucleotide divergence (D_{XY})

To estimate global per-site D_{XY} values from the WGR data, allele frequencies for all individuals were estimated with ANGSD, assuming fixed major and minor alleles (-doMaf 1). Sites with p-values >1×10^{-6} (-SNP_pval 1e-6) and triallelic sites were removed (-skipTriallelic 1). Major and minor alleles were inferred from genotype likelihoods (doMajorMinor 1) using the SAMTOOLS model (-GL 1). Next, we reran the allele frequencies per population with the -site flag with indexed sites recovered from the previous step. Unzipped maf files generated for each population were then used to calculate the global per-site D_{xy} value using the accessory Rscript (calcDxy.R) in NGSTOOLS (20). For context, we estimated nucleotide divergence among related pairs of species. The eight *C. diabolis* and thirteen *C. nevadensis* samples used in Tian et al. (21) were downloaded and aligned to the non-repetitive unambiguous regions within the *C. brontotheroides* reference genome (GCA_018398635.1) and analyzed per methods above.

Demographic modeling

To create a demographic model of the three *C. tularosa* populations (MS, SC, LR), the observed joint 3D-SFS was first estimated using ANGSD v.0.930. The folded 1D-SFS of each population was individually estimated using genotype likelihoods of all sites with a minimum mapping quality score of 35 *(*-dosaf 1 -minQ 35 -GL 1). The SFS of the three populations was then combined using the realSFS package within ANGSD to obtain the observed 3D-SFS. To identify the most likely demographic model, GADMA v. 2.0.0rc20 (22) was used to simulate the SFS of 3 populations under different demographic scenarios using the ∂a∂i diffusion approximation (23) and comparing it to the observed SFS. For the simulations, a point mutation rate of $\mu = 6.6 \times 10^{-8}$ found in cichlids (24) and a generation time of 1 year was used, keeping all other parameters to default values. We also set the migration to false, to reflect the lack of gene flow between the two ESUs since the lava flow isolated each population (25, 26), which is corroborated by the high F_{ST} values we observed. For each demographic model, GADMA simulates the joint SFS and compares it to the observed SFS using log-likelihoods and the most likely model is chosen using AIC score for all models. To estimate 95% confidence intervals (CI) around the mean estimates of population size (N_e) and divergence times (T) from the most likely demographic model, the observed joint SFS was bootstrapped 100 times using independent subsets of SNPs. To create sets of unlinked SNPs, we re-

estimated SFS by sampling only 1 SNP from each scaffold (i.e., total number of unliked SNPs used for bootstrapping=1,285).

The two main uncertainties with this type of modeling are the estimates of generation time and mutation rate. We employed a generation time of one year (effectively an annual species) as there are no documented estimates available for *C. tularosa*. However, desert fishes often reach maturity in several months and breed during Spring and Summer, so can have more than one generation per year (27). This means that the estimated divergence time between ESUs likely occurred between 4-8kya. There is controversy in the literature about the mutation rate in Cyprinodontidae (28, 29). Therefore, we used the best available estimate from the literature, 6.6×10^{-8} mutations per site per year, as determined from a pedigree analysis conducted using a cichlid F_2 intercross (24). This mutation rate had been previously utilized in the context of the Devil's Hole divergence time analysis by Martin and Höhna (28). We think our mutation rate estimate and generation time estimates are relatively robust given the general agreement between our resulting demographic model and the known demographic history of the translocation from SC to LR (48 years historical documentation vs 59 years estimated here).

Tree-based analyses

A *bcf* file was generated for all 45 WGR individuals from genotype likelihoods implemented in ANGSD (-dobcf), and variant calls (n=215,846 nt) for each sample were extracted and concatenated using VCF-KIT (31) before running IQ-TREE v.2.1.2 (32) on variant sites with the GTR+ASC model. To prevent overestimating branch lengths, the ascertainment bias correction (+ASC) model was used. Branch support of the tree was evaluated by both ultrafast bootstrap (-B) approximation and SH-like approximate likelihood ratio test (-alrt) with 1,000 bootstraps for each. Sequence Read Archive files were downloaded for *C. variegatus* (SRX526475), processed as above, and were included as the outgroup.

To evaluate the *C. tularosa* matrilineal patterns of population differentiation, mitochondrial sequences were assembled and compared. Complete mitogenomes for all 45 WGR individuals were reconstructed using the quality filtered Illumina paired-end reads. COALQC v.0.1 (33) and SAMTOOLS v.1.17 were used to extract Illumina reads that aligned to the NCBI *C. tularosa* mitochondrial genome (NC_028292.1). The resulting *bam* files were then converted back to *fastq* file format with BEDTOOLS v.2.30.0 and used with the NCBI reference to guide individual mitochondrial genome assemblies with MITOBIM v.1.8 (34).

All reconstructed mitogenomes, along with 11 other *Cyprinodon* mitogenomes, plus one mitogenome for *C. tularosa,* were downloaded from NCBI (all *Cyprinodon* mitogenomes available to date) and subjected to multiple sequence alignment using the default parameters employed by CLUSTALX v.2.1 (35). Two

maximum likelihood phylogenies were inferred using IQ-TREE with TIM3+F+I+G4 as the best fit model. The first phylogeny was based on the alignment of mitogenomes from 45 *C. tularosa* in this study, along with mitogenomes of *C. tularosa* and *C. variegatus* available on NCBI where *C. variegatus* (NC_028088.1) was used as the outgroup (Fig. 4*B*). The second mitochondrial tree encompassed all 45 newly generated mitogenomes and 12 *Cyprinodon* mitogenomes retrieved from NCBI where *C. julimes* (MG727890.1) was considered as the outgroup (Fig. S6). As above, branch support of the tree was evaluated by both ultrafast bootstrap (-B) approximation and SH-like approximate likelihood ratio test (-alrt) with 1000 bootstraps for each.

Coalescent-based species delimitation and species tree analysis

Species delimitation was assessed using BAYESIAN PHYLOGENETICS AND PHYLOGEOGRAPHY BPP v.4.6.2 (36, 37). This method uses the multispecies coalescence to compare different models of species delimitation (38, 39) and species phylogeny (40, 41) in a Bayesian framework, accounting for incomplete lineage sorting due to ancestral polymorphism and gene tree species discordance. Two analyses A10 (species delimitation using a user-specified guide tree) and A11 (joint species delimitation and species tree inference using unguided species delimitation) were employed. We considered MS, SC, and LR as three distinct populations in the A11 BPP analysis. We used the species tree generated from IQ-TREE, (MS, LR/SC) as the guide tree in the A10 analysis. Due to the large computational footprint involved with these analyses, BPP inferences were performed on a reduced dataset comprised of 1,000 nuclear DNA (nDNA) loci. We also separately conducted BPP analyses on the complete mitogenomes of 45 individuals, considering the whole mitogenome (mtDNA) as a single locus.

For the selection of nDNA loci, BCFTOOLS v.1.8 was used for variant calling. Sites were removed if they had a genotype quality below 20 (GQ<20), a minimum depth below 5 (DP<5), and a maximum depth greater than 20 (DP>20). Sites that did not meet these filtering thresholds were hard masked and all insertions and deletions were replaced with dashes. Extracted filtered genotypes were then converted to sequences using the BCFTOOLS consensus module, with heterozygous sites represented using the International Union of Pure and Applied Chemistry (IUPAC) codes. We selected 1,000 loci by implementing the following filtering criteria on the generated sequences to satisfy the following BPP assumptions: (i) the locus length should be between 500-1,000 nucleotides to ensure no recombination within a locus, (ii) less than 5% missing data per locus, (iii) each locus should belong to a separate scaffold to ensure enough physical distance to assume free recombination between loci. We followed the BPP authors recommendation, in that multiple genes from the mitochondrial genome should be treated as a single locus in the MSC-based analysis, which generally does not undergo recombination. Ultimately, we considered the whole mitogenome as a single locus and ran each of the 1,000 nuclear loci separately in the BPP analyses.

We used the JC69 substitution model, as this is adequate for closely related species and used equal probabilities for the species model prior (speciesmodelprior 1). The analysis was run with both the algorithms 0 and 1 with different values of ε and α and m, respectively, and different seed numbers. For each species tree model, we assign the inverse-gamma priors θ~IG (3, 0.003) for all θs and τ ~ IG (3, 0.002) for the age τ0 of the root. To establish the priors, a diffuse prior (a = 3) was employed, and b was adjusted to ensure a reasonable mean. For the parameter theta, which quantifies genetic diversity (heterozygosity) within a species, the prior was set based upon the calculated genetic diversity in our samples. We set a sensible theta prior of (3) 0.003 based upon the estimated heterozygosity in our samples, with the mean 0.003/(3-1)=0.0015 (a difference of 1.5 per kb). The tau prior was set to (3) 0.002, with the sequence divergence mean of 0.001 (1 difference per kb). The analysis consisted of 200,000 MCMC iterations after a burn-in period of 10,000 iterations, with sampling occurring every two iterations (for a total of 100,000 samples). The step lengths used in the MCMC proposals were adjusted automatically (using the finetune parameters) to stay within the 20-70% range. Results were compared between runs to check for convergence. As reported in previous studies and BPP documentation, the program may result in mixing issues with large data sets. To test the robustness of our results, we randomly selected five sets of nDNA loci, each comprising 20 loci (total of 100 loci), and separately carried out A11 analyses employing both rj0 and rj1 algorithms. The priors were set to θ~ G (3, 2000) and τ ~ G (3, 2000) with all the other parameters consistent with the above analyses.

We sought to compare the demographic results obtained from GADMA by conducting a demography and divergence time estimation analysis (A00) in BPP using five sets of 20 nuclear loci (100 total loci), with three replicates of each. We used the same 100 nuclear loci and priors as the A11 analysis above. Parameterization included a generation time of one year, a mutation rate of 6.6×10^{-8} mutations per site per year, and we used a fixed species tree (MS, SC). Results indicated the ancestral MS/SC divergence occurred 924 -2,444 years ago (95% CI=833 -3318). Estimated effective population sizes (*Ne)* from the model were 1,333-1,939 (95% =462-3,196) for MS and 901- 1,503 (95%CI= 272-2,454) for the SC population (*SI Appendix*, Table S2).

Detection of selection signatures between ESUs

To identify signatures of selection between the two ESUs, the SC+LR samples were collapsed into one population (ESU2) and compared to the MS (ESU1). Due to potential issues associated with merging the SC+LR pool-seq libraries, analysis on putative genes under selection was carried out for the WGR dataset only (i.e., not for the pool-seq data). Weighted pairwise F_{ST} of quality filtered sites was estimated between ESUs using ANGSD. First, site allele frequency likelihoods were estimated for each ESU, followed by the generation of folded 2D site

frequency spectra (realSFS). The average pairwise weighted F_{ST} (realSFS fst) was then calculated. To assess the genome-wide patterns of genetic differentiation, we employed a 50kb non-overlapping sliding window approach to calculate F_{ST} values using ANGSD (realSFS fst stats2). Any windows containing less than 20,000 sites were excluded from subsequent analyses. The genome wide F_{ST} values around the mean for each sliding window were then arranged in descending order, and the top 2.5% of these values were chosen as the threshold (F_{ST}=0.885) following Bahbahani et al. (42).

We compiled an *a priori* list of 34 candidate genes from a previous study on transcriptional dynamics of ion transporters and aquaporins in the gill of the desert Amargosa pupfish (*C. nevadensis amargosae*) during rapid salinity change (43); the list includes osmoregulatory genes, aquaporins, solute carriers and ion co-transporters, as well as the *pgd* gene encoding an allozyme previously examined in *C. tularosa*. Because of the considerable salinity differences in MS and SC habitats, we expected these genes to exhibit high differentiation and be positioned within the top 2.5% of the F_{ST} distribution, indicating strong divergent selection. To further analyze these candidate genes, we estimated pairwise F_{ST} and Tajima's D values in non-overlapping 5kb windows for the identified F_{ST} peaks containing the candidate genes within the top 2.5% threshold. To assess the patterns of absolute genetic differentiation within the identified genes falling within the top 2.5% threshold, we calculated D_{XY} on a per-SNP basis using the *calcDxy.R* script.

Evaluation of previous utilized allozymes

Genes underlying the allozyme proteins previously showing variation among populations (44, 45) were re-examined using both pool-seq and WGR data by extracting the genic coordinates from the *C. tularosa* reference genome and performing a principal component analysis on the allele frequencies within each genic region. While the phosphogluconate dehydrogenase (*pgd)* gene (Fig. S7) showed substantial variation among the three populations, there was no clear pattern of clustering that corresponded to the ESUs. The glucokinase / hexokinase-4 (*gck*) gene was previously shown to have fixed or nearly fixed differences when comparing MS to the other populations (45), but this pattern was not observed in our genomic datasets; neither pool-seq or WGR data identified any variable sites within this gene. We repeated our methods using unfiltered alignment files (in the event that some *gck* variants were inadvertently filtered out), but all sites within this gene remained invariant. This suggests to us that the *gck* polymorphisms observed by Stockwell and Mulvey (44) at the protein level may been due to RNA editing, alternative splicing, post-translational modifications, or some other form of gene expression that gave rise to distinct polypeptides in the two different environments of MS vs SC/LR.

Estimating genetic load

As small, isolated populations are prone to heavy genetic load (i.e., an abundance of fixed deleterious mutations), we evaluated load within each population. We created a database using the annotated *C. tularosa* reference genome and lethality of variants was predicted using SNPEFF (46). This assigns a categorial impact class to each variant, defined as high impact (e.g., loss of function), moderate impact, low impact, and no-impact (e.g., in noncoding regions). The intersection between high-impact variants among populations was evaluated using a Venn diagram to infer likely ancestral and derived mutations (Fig. S4). The annotated SNPs were further examined to determine the ratio of non-synonymous mutations (Dn) to synonymous mutations (Ds) for the coding sequences of the 34 candidate genes. However, only 4/34 of the coding sequences (aqp3a, ca6, slc4a5b, slc4a2a) harbored several variants; none were found for MS, two non-synonymous and one synonymous were found for SC, and two synonymous mutations were found for LR.

SI References

1. A. N. Black, J. R. Willoughby, A. Brüniche-Olsen, B. L. Pierce, J. A. DeWoody, The endangered White Sands pupfish (*Cyprinodon tularosa*) genome reveals low diversity and heterogenous patterns of differentiation. *Mol. Ecol. Resour.* **21**, 2520–2532 (2021).
2. C. Pockrandt, M. Alzamel, C. S. Iliopoulos, K. Reinert, GenMap: ultra-fast computation of genome mappability. *Bioinformatics* **36,** 3687–3692 (2020).
3. A. R. Quinlan, I. M. Hall, BEDTools: a flexible suite of utilities for comparing genomic features. *Bioinformatics* **26,** 841–842 (2010).
4. P. Ewels, M. Magnusson, S. Lundin, M. Käller, MultiQC: summarize analysis results for multiple tools and samples in a single report. *Bioinformatics* **32,** 3047–3048 (2016).
5. S. J. Micheletti, S. R. Narum, Utility of pooled sequencing for association mapping in nonmodel organisms. *Mol. Ecol. Resour.* **18,** 825–837 (2018).
6. H. Li, Aligning sequence reads, clone sequences and assembly contigs with BWA-MEM. arXiv [Preprint] (2013). https://arxiv.org/abs/1303.3997v2 (accessed 01/06/2022).
7. G. G. Faust, I. M. Hall, SAMBLASTER: fast duplicate marking and structural variant read extraction. *Bioinformatics* **30,** 2503–2505 (2014).
8. H. Li *et al.,* The sequence alignment/map format and SAMtools. *Bioinformatics* **25,** 2078–2079 (2009).
9. H. Li, A statistical framework for SNP calling, mutation discovery, association mapping and population genetical parameter estimation from sequencing data. *Bioinformatics* **27,** 2987–2993 (2011).

10. R. Kofler, R. V. Pandey, C. Schlötterer, PoPoolation2: identifying differentiation between populations using sequencing of pooled DNA samples (Pool-Seq). *Bioinformatics* **27,** 3435–3436 (2011).
11. A. M. Bolger, M. Lohse, B. Usadel, Trimmomatic: a flexible trimmer for Illumina sequence data. *Bioinformatics* **30,** 2114–2120 (2014).
12. A. McKenna *et al.,* The Genome Analysis Toolkit: a MapReduce framework for analyzing next-generation DNA sequencing data. *Genome Res.* **20,** 1297–1303 (2010).
13. GA Van der Auwera, From FastQ data to high confidence variant calls: the Genome Analysis Toolkit best practices pipeline. *Curr. Protoc. Bioinformatics* **43,** 11.10.1–11.10.33 (2013).
14. T. S. Korneliussen, A. Albrechtsen, R. Nielsen, ANGSD: analysis of next generation sequencing data. *BMC Bioinformatics* **15,** 1–13 (2014).
15. J. Meisner, A. Albrechtsen, Inferring population structure and admixture proportions in low-depth NGS data. *Genetics* **210**, 719–731 (2018).
16. H. Wickham, *ggplot2: Elegant Graphics for Data Analysis* (Springer-Verlag New York, 2016).
17. L. Skotte, T. S. Korneliussen, A. Albrechtsen, Estimating individual admixture proportions from next generation sequencing data. *Genetics* **195,** 693–702 (2013).
18. R. M. Francis, pophelper: an R package and web app to analyse and visualize population structure. *Mol. Ecol. Resour.* **17,** 27–32 (2017).
19. G. Evanno, S. Regnaut, J. Goudet, Detecting the number of clusters of individuals using the software STRUCTURE: a simulation study. *Mol. Ecol.* **14,** 2611–2620 (2005).
20. M. Fumagalli, F. G. Vieira, T. Linderoth, R. Nielsen. ngsTools: methods for population genetics analyses from next-generation sequencing data. *Bioinformatics* **30,** 1486–1487 (2014).
21. D. Tian, A. H. Patton, B. J. Turner, C. H. Martin, Severe inbreeding, increased mutation load and gene loss-of-function in the critically endangered Devils Hole pupfish. *Proc. R. Soc. B.* **289,** 20221561 (2022).
22. E. Noskova, V. Ulyantsev, K.-P. Koepfli, S. J. O'Brien, P. Dobrynin, GADMA: Genetic algorithm for inferring demographic history of multiple populations from allele frequency spectrum data. *Gigascience* **9,** giaa005 (2020).
23. R. N. Gutenkunst, R. D. Hernandez, S. H. Williamson, C. D. Bustamante, Inferring the joint demographic history of multiple populations from multidimensional SNP frequency data. *PLoS Genet.* **5,** e1000695 (2009).
24. H. Recknagel, K. R. Elmer, A. Meyer, A hybrid genetic linkage map of two ecologically and morphologically divergent Midas cichlid fishes

(*Amphilophus* spp.) obtained by massively parallel DNA sequencing (ddRADSeq). *G3 (Bethesda)* **3**, 65–74 (2013).

25. R. R. Miller, A. A. Echelle, *Cyprinodon tularosa*, a new Cyprinodontidae fish from the Tularosa Basin, New Mexico. *Southwest Nat*. **19,** 365–377 (1975).
26. J. S. Pittenger, C. L. Springer, Native range and conservation of the White Sands pupfish (*Cyprinodon tularosa*). *Southwest Nat*. **44**, 157–165 (1999).
27. G. D. Constantz, “Life history patterns of desert fishes” *in Fishes in North American deserts*, R. J. Naiman, D. L. Soltz, Eds. (Wiley, 1981), pp. 237–290.
28. C. H. Martin, S. Höhna, New evidence for the recent divergence of Devil's Hole pupfish and the plausibility of elevated mutation rates in endangered taxa. *Mol. Ecol.* **27**, 831–838 (2017).
29. İ. K. Sağlam et al., Phylogenetics support an ancient common origin of two scientific icons: Devils Hole and Devils Hole pupfish. *Mol. Ecol*. **25,** 3962–73 (2016).
30. P. Danecek et al., Twelve years of SAMtools and BCFtools. *Gigascience* **10**, giab008 (2021).
31. D. E. Cook, E. C. Andersen, VCF-kit: assorted utilities for the variant call format. *Bioinformatics* **33,** 1581–1582 (2017).
32. B. Q. Minh, IQ-TREE 2: new models and efficient methods for phylogenetic inference in the genomic era. *Mol. Biol. Evol.* **37,** 1530–1534 (2020).
33. A. B. Patil *et al*., CoalQC-Quality control while inferring demographic histories from genomic data: Application to forest tree genomes. bioRxiv. [Preprint] (2020). https://www.biorxiv.org/content/10.1101/2020.03.03.962365v1.full. (accessed 20 September 2022).
34. C. Hahn, L. Bachmann, B. Chevreux, Reconstructing mitochondrial genomes directly from genomic next-generation sequencing reads—a baiting and iterative mapping approach. *Nucleic Acids Res.* **41,** e129 (2013).
35. M. A. Larkin *et al.,* Clustal W and Clustal X version 2.0. *Bioinformatics* **23,** 2947–2948 (2007).
36. Z. Yang, The BPP program for species tree estimation and species delimitation. *Curr. Zool*. **61**, 854–865 (2015).
37. T. Flouri, X. Jiao, B. Rannala, Z. Yang, Species tree inference with BPP using genomic sequences and the multispecies coalescent. *Mol. Biol. Evol.* **35**, 2585–2593 (2018).

38. Z. Yang, B. Rannala, Bayesian species delimitation using multilocus sequence data. *Proc. Natl. Acad. Sci. U.S.A.* **107**, 9264–9269 (2010).
39. B. Rannala, Z. Yang, Improved reversible jump algorithms for Bayesian species delimitation. *Genetics* **194**, 245–253 (2013).
40. Z. Yang, B. Rannala, Unguided species delimitation using DNA sequence data from multiple loci. *Mol. Biol. Evol.* **31**, 3125–3135 (2014).
41. B. Rannala, Z. Yang, Efficient Bayesian species tree inference under the multispecies coalescent. *Syst. Biol.* **66,** 823–842 (2017).
42. H. Bahbahani *et al.,* Signatures of positive selection in East African Shorthorn Zebu: A genome-wide single nucleotide polymorphism analysis. *Sci Rep*. **5,** 11729 (2015).
43. S. C. Lema, P. G. Carvalho, J. N. Egelston, J. T. Kelly, S. D. McCormick, Dynamics of gene expression responses for ion transport proteins and aquaporins in the gill of a euryhaline pupfish during freshwater and high-salinity acclimation. *Physiol. Biochem. Zool*. **91,** 1148–1171 (2018).
44. C. A. Stockwell, M. Mulvey, A. G. Jones, Genetic evidence for two evolutionarily significant units of White Sands pupfish. *Anim. Conserv.* **1,** 213–225 (1998).
45. C. A. Stockwell, M. Mulvey, Phosphogluconate dehydrogenase polymorphism and salinity in the White Sands pupfish. *Evolution* **52**, 1856–1860 (1998).
46. P. Cingolani *et al.,* A program for annotating and predicting the effects of single nucleotide polymorphisms, SnpEff: SNPs in the genome of Drosophila melanogaster strain w1118; iso-2; iso-3. *Fly* **6,** 80–92 (2012).

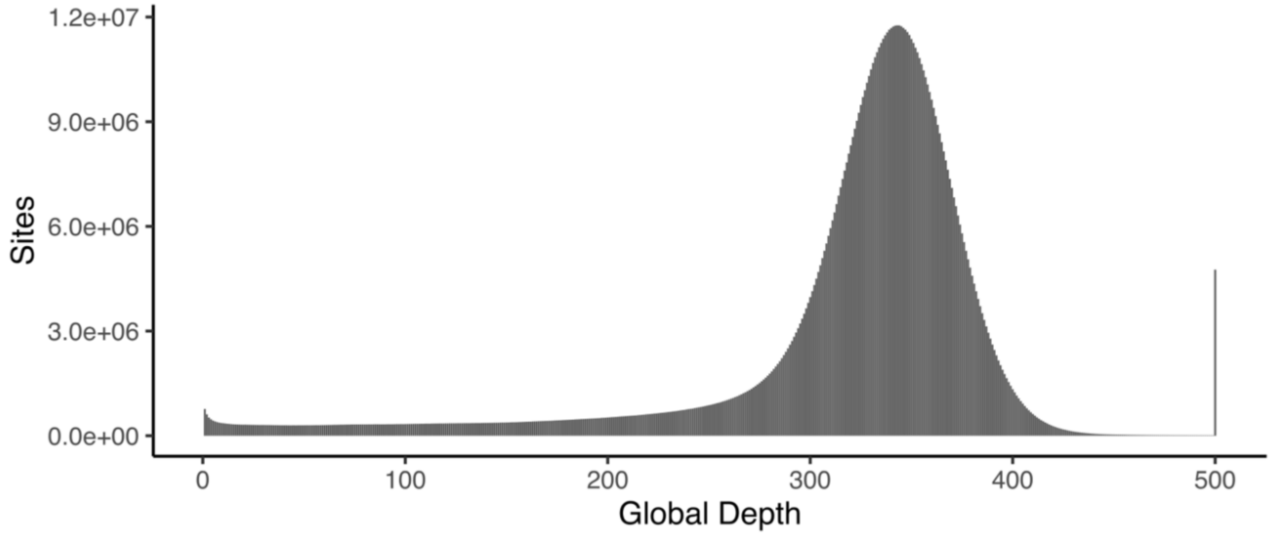

Fig. S1. *Global coverage distribution.* The number of sites at each depth of coverage among all whole genome resequencing samples.

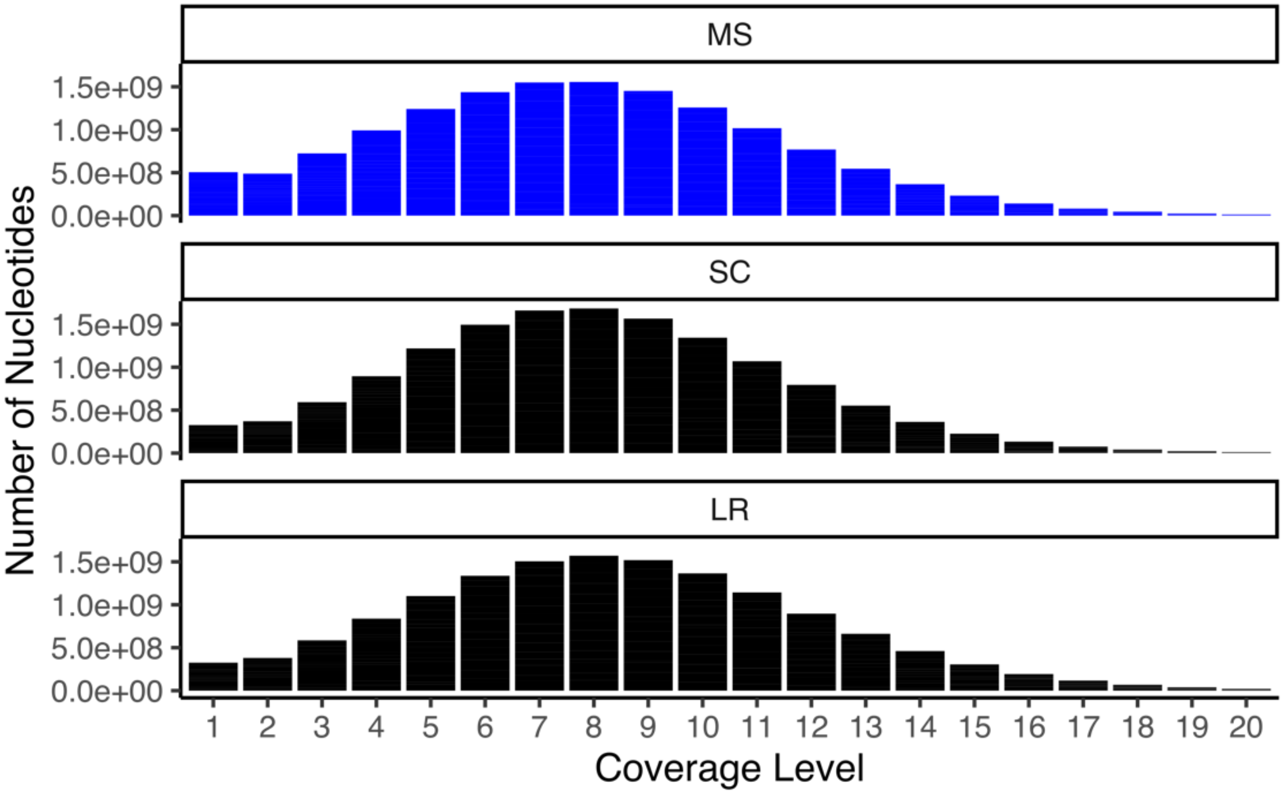

Fig. S2. *Global coverage by population.* Coverage for all 45 *C. tularosa*, parsed by sampling sites among whole genome resequencing samples and colored according to the Evolutionarily Significant Unit (ESU) the population is assigned to (Malpias Spring ESU1=Blue, Salt Creek+Lost River ESU2=Black).

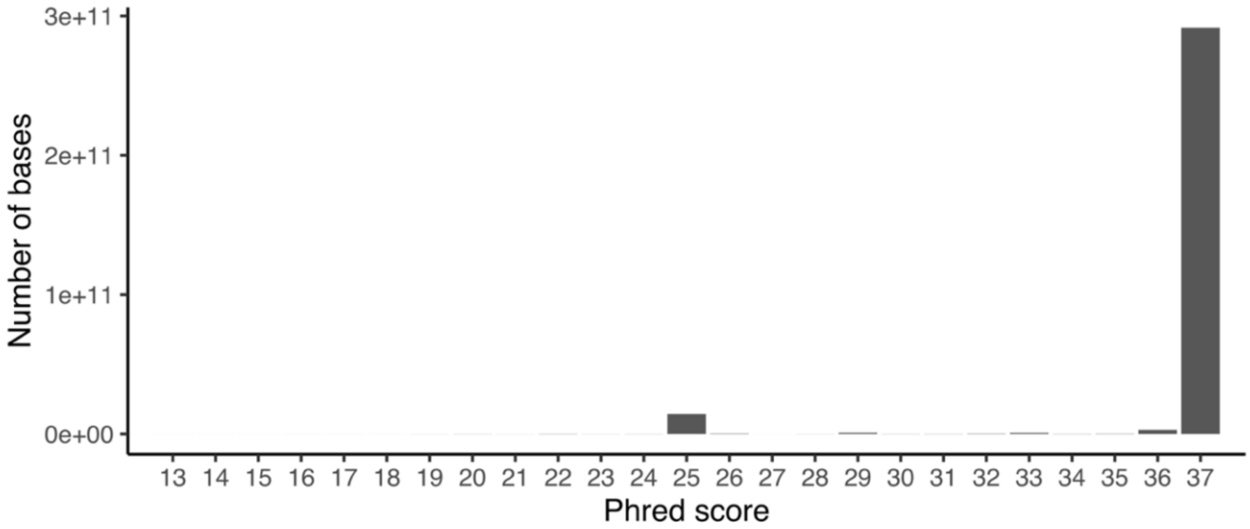

Fig. S3. *Phred score distribution.* Histogram of base quality scores among all reads derived from whole genome resequencing prior to any filtering.

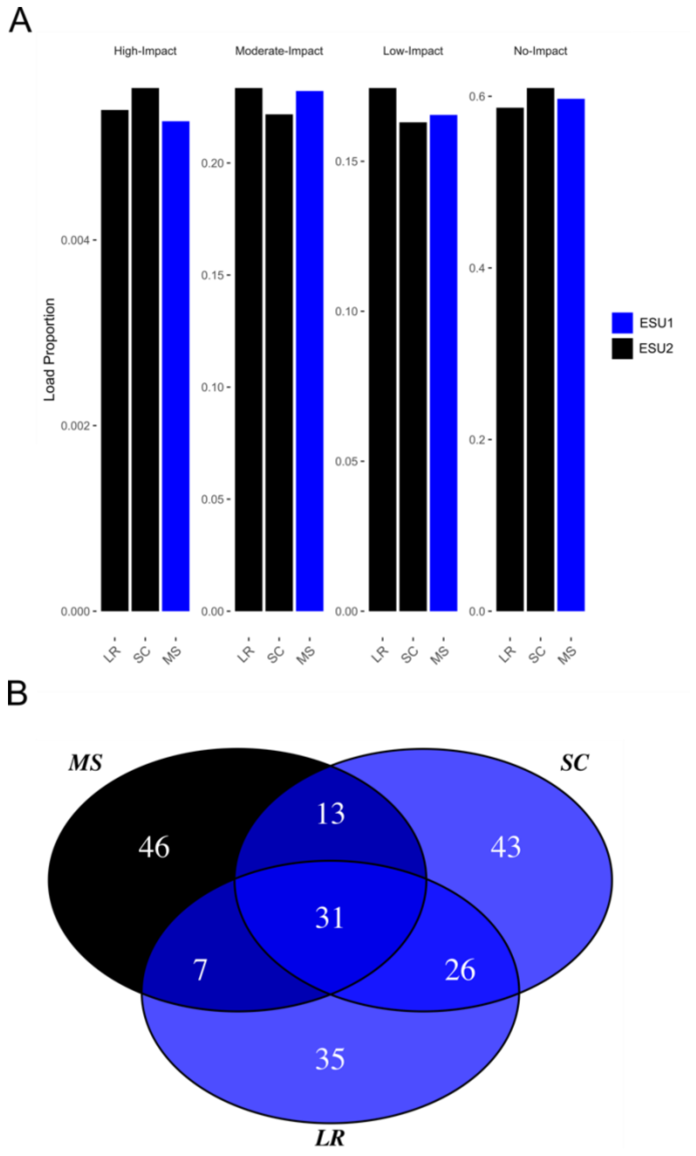

Fig. S4. *Evaluation of genetic load within each C. tularosa population.* (A) The proportion of total load within each population, categorized by predicted impact class (high, moderate, low) of deleterious variants. Note, the different scales for each class (B) The intersection of high impact variants found within each population. Overall, 31 high impact mutations were shared (likely ancestral) and a range (35-46) were found in isolation (likely derived mutations). As theory predicts, LR had the lowest number of deleterious variants, likely due the historical bottlenecks followed by the documented founding event that occurred in 1970s. Fields are colored according to the Evolutionarily Significant Unit (ESU) the population is assigned to (Malpias Spring ESU1=Blue, Salt Creek+Lost River ESU2=Black).

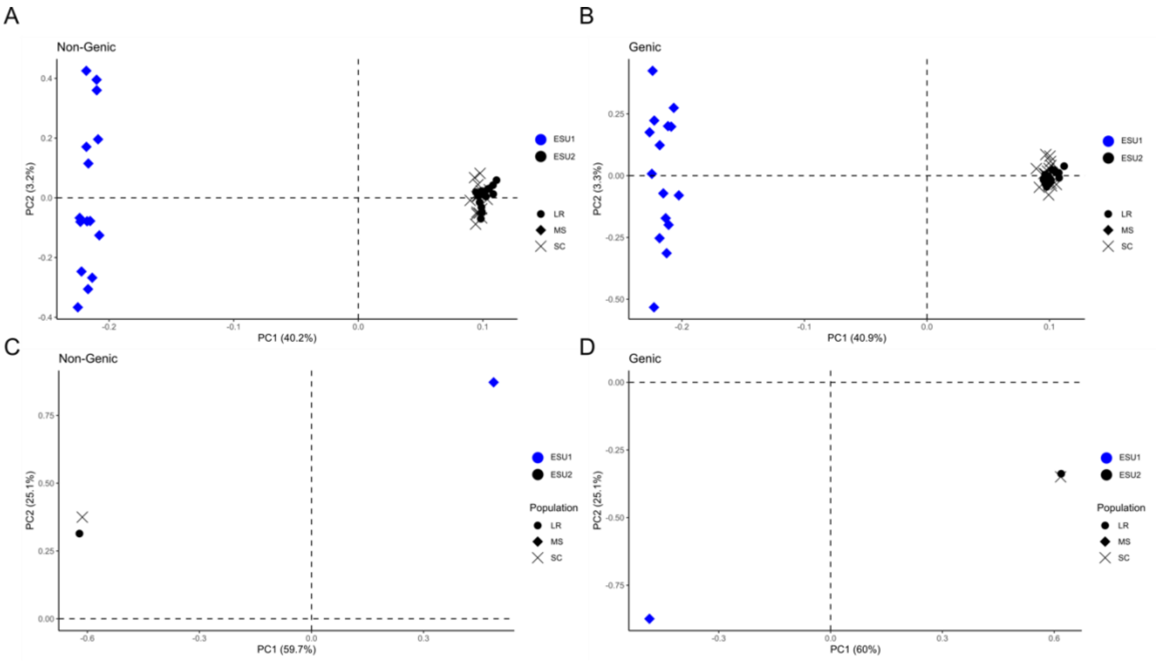

Fig. S5. *Principal component analysis for sites found in Non-Genic or Genic regions.* Principal component analysis of allele frequencies (maf>0.05) derived from whole genome resequencing (A-B) and pool-seq (C-D) data. Colors represent the two *C. tularosa* Evolutionary Significant Units (ESUs) and shapes represent sampling locations for individuals collected from LR (Lost River), MS (Malpais Spring), and SC (Salt Creek). Variation explained for the first two components is labeled on each axis for each sequencing approach/genomic region.

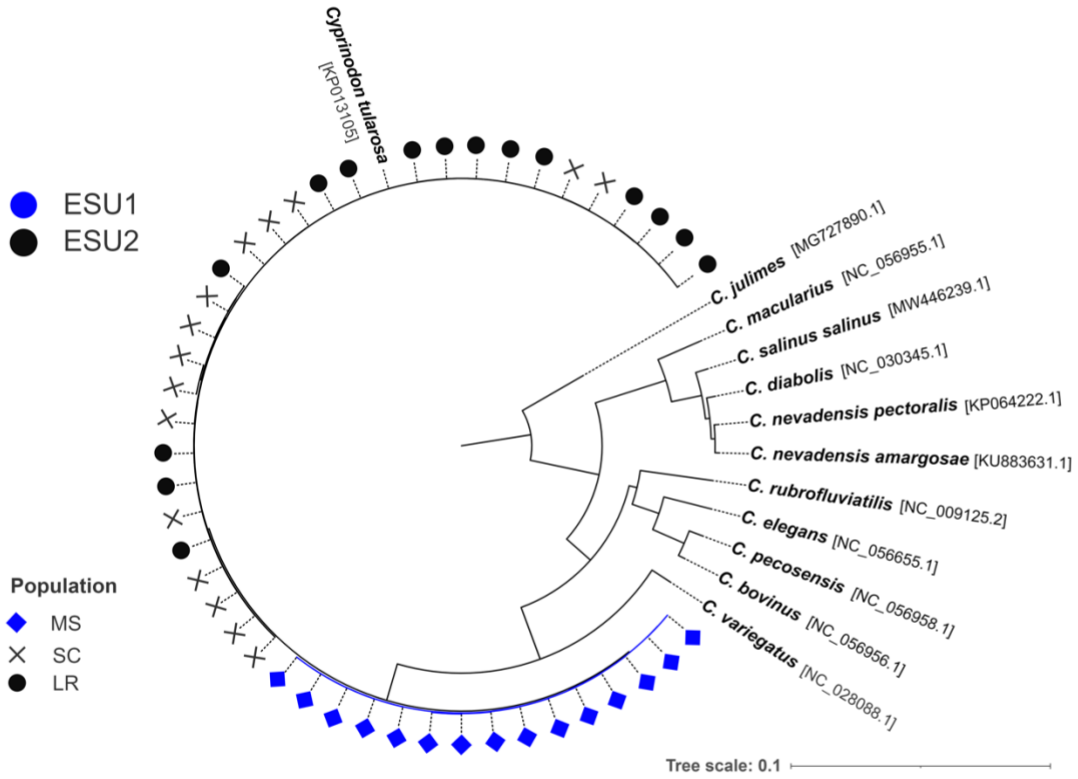

Fig. S6. *Maximum likelihood tree of Cyprinodontidae mitochondrial genomes*. Tree derived from the complete mitogenomes of 45 *C. tularosa* used in this study and twelve other pupfish species (including an additional *C. tularosa* from NCBI). Points are colored according to the Evolutionarily Significant Unit (ESU) the population is assigned to (Malpias Spring ESU1=Blue, Salt Creek+Lost River ESU2=Black).

0.008

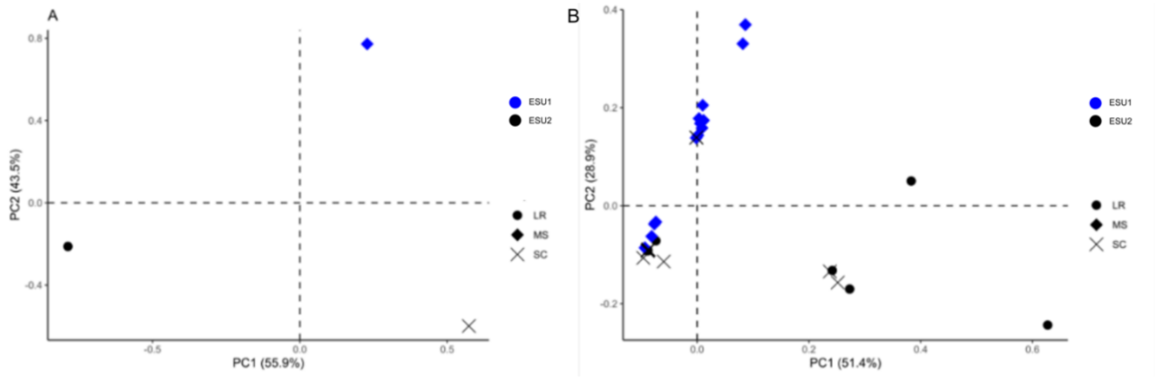

Fig. S7. Principal component analysis of the phosphoguluconate dehydrogenase gene *(pgd).* PCA of allele frequencies (maf>0.01) generated from pool-seq (A) and whole genome resequencing data (B) using sites found within the *pgd* gene. Variation explained is labeled on each axis for each sequencing method.

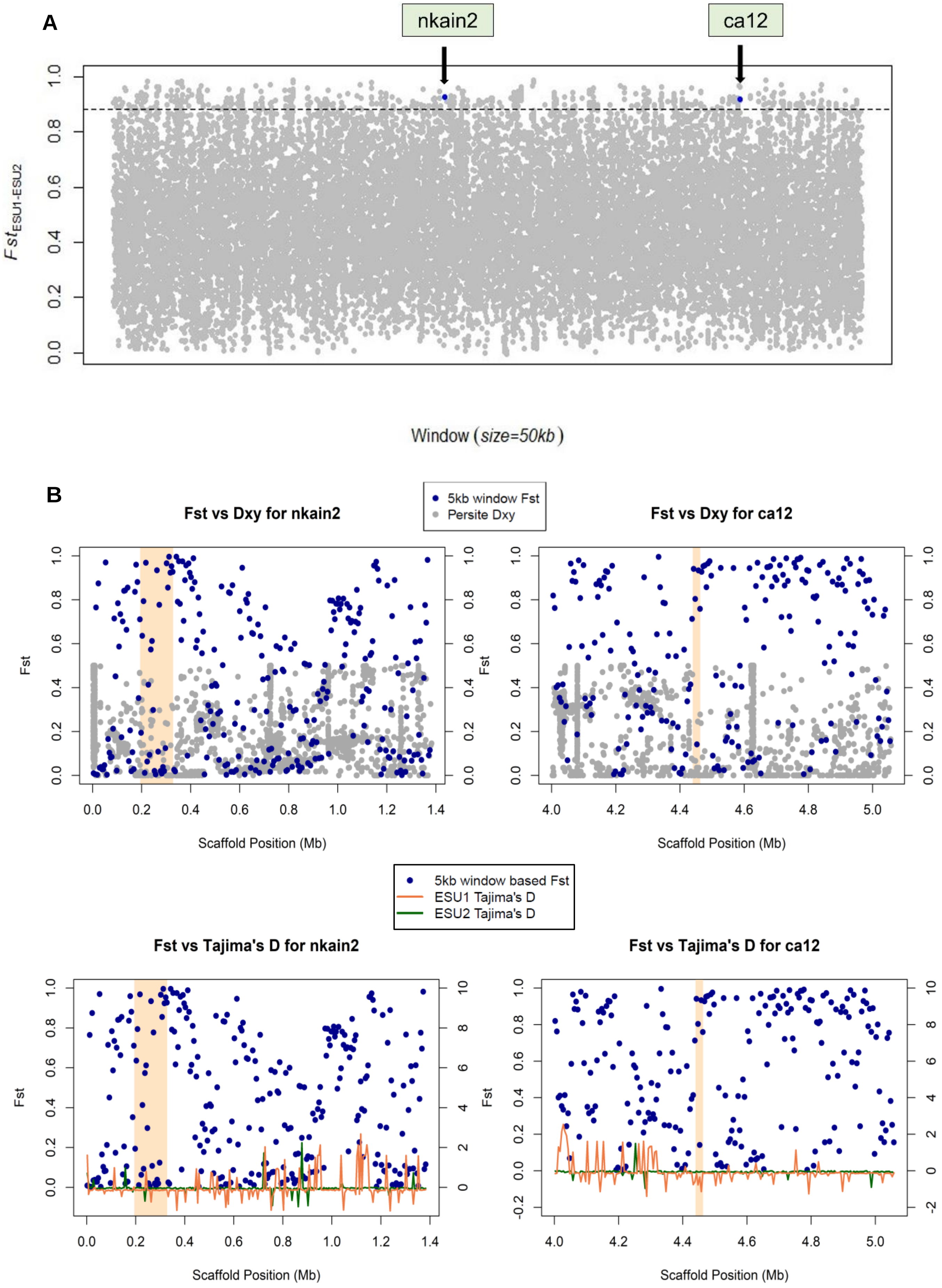
A
nkain2
ca12
$Fst_{ESU1-ESU2}$
Window (size=50kb)
B
5kb window Fst
Persite Dxy
Fst vs Dxy for nkain2
Fst vs Dxy for ca12
Fst
Scaffold Position (Mb)
5kb window based Fst
ESU1 Tajima's D
ESU2 Tajima's D
Fst vs Tajima's D for nkain2
Fst vs Tajima's D for ca12
Fst
Scaffold Position (Mb)

Fig. S8. *Detecting signatures of local adaptation.* Genome-wide sliding window analysis (window size=50kb) for $F_{ST.}$ (A) The dashed line indicates the top 2.5% threshold for F_{ST} (0.885). Windows containing an *a priori* candidate gene(s) identified in *C. tularosa* genome are colored in blue. Two of these windows contained targeted genes within the top 2.5% F_{ST} windows; *nkain2* and *Ca12*. To further assess these regions, pairwise F_{ST} was re-estimated in 5kb windows for the identified outlier genes (*nkain2* and *ca12*) and overlaid with per-site absolute divergence (D_{XY}) between ESUs (Malpais Spring= ESU1, Salt Creek+Lost River=ESU2). Also, Tajima's *D* was estimated in 5kb windows and displayed for each ESU (B). Vertical highlighted bars represent outlier candidate gene regions, *nkain2* and *Ca12*, in each plot.

Table S1. Parameters estimates of the most likely complex 3-population demographic model with no migration based on composite likelihood method. The 95% confidence intervals were estimated from 100 bootstrapped SFS estimated from unlinked SNPs.

No. of parameters estimated	13
Log likelihood	-1932218.59
Divergence time in years [95 % CI]	
T_1	4,153.67 [3,871.43 – 4,435.84]
T_2	2,475.02 [2,262.81 – 2687.13]
T_3	58.78 [48.12 – 69.44]
Split ratios [95% CI]	
$S_{p11:p12}$	0.953 [0.948 – 0.958]
$S_{SC:LR}$	0.901 [0.872 – 0.930]
Population size [95% CI]	
N_{anc}	3,911 [3,644 – 4,178]
N_{11}	64,916 [61,893 – 67,939]
N_{12}	17,070 [14,891 – 19,249]
N_{21}	69 [58 – 80]
N_{22}	193 [168 – 218]
N_{LR}	698 [578 – 818]
N_{SC}	8,714 [8,196 – 9,232]
N_{MS}	182 [166 – 198]

Table S2. Summary of Bayesian Phylogenetics and Phylogeography demography and divergence time estimation results. Five sets of 20 different nuclear loci (100 total loci) were analyzed using priors of θ~ G (3, 2000) and τ ~ G (3, 2000). The θs and T parameter estimates are averages of three replicates for each set of loci; reporting mean values with associated 2.5 and 97.5 percentiles of the posterior distributions for each set of loci. *Ne*=Effective population size, MS=Malpais Spring population, SC=Salt Creek population.

**loci set**	**Mean, 2.5 and 97.5 percentiles of the estimate**	**Average parameter estimates**			***Ne***		**Divergence time (years)**
		**theta MS**	**Theta SC**	**tau**	**MS**	**SC**	
**1**	mean	0.000425	0.000327	0.000128	1,609	1,238	1,944
	2.5	0.000187	0.000131	0.000119	708	496	1,803
	97.5	0.000831	0.000641	0.000174	3,147	2,428	2,636
**2**	mean	0.000364	0.000238	0.000161	1,378	901	2,444
	2.5	0.000140	0.000078	0.000155	530	295	2,348
	97.5	0.000729	0.000503	0.000219	2,761	1,905	3,318
**3**	mean	0.000512	0.000397	0.000061	1,939	1,503	924
	2.5	0.000290	0.000227	0.000055	1,098	859	833
	97.5	0.000844	0.000648	0.000076	3,196	2,454	1,151
**4**	mean	0.000352	0.000239	0.000143	1,333	905	2,171
	2.5	0.000122	0.000072	0.000135	462	272	2,045
	97.5	0.000731	0.000539	0.000196	2,768	2,041	2,974
**5**	mean	0.000382	0.000268	0.000088	1,446	1,015	1,328
	2.5	0.000143	0.000091	0.000082	541	344	1,242
	97.5	0.000852	0.000594	0.000124	3,227	2,250	1,883

Table S3. Global pairwise F_{ST} estimates (±standard deviation) for pool-seq (above diagonal) and Whole Genome Resequencing (below diagonal) datasets for the three focal pupfish populations among the two Evolutionarily Significant Units (ESU).

	ESU1	ESU2	
	Malpais Spring	Salt Creek	Lost River
Malpais Spring	-	0.282(±0.250)	0.275(±0.250)
Salt Creek	0.406(±0.244)	-	0.046(±0.078)
Lost River	0.400(±0.251)	0.142(±0.175)	-

Table S4. Summarized species delimitation results of BPP analyses A11 and A10 using different datasets, algorithms, and parameters. Posterior probability values (P[N]) are reported for each run.

**Analysis**	**Locus/ loci**	**algorithm0**			**algorithm1**		
		**speciesdelimitation= 1 0 2**	**speciesdelimitation= 1 0 5**	**speciesdelimitation= 1 0 10**	**speciesdelimitation= 1 1 2 2**	**speciesdelimitation= 1 1 2 1**	**speciesdelimitation= 1 1 1 5**
**A11**	mtDNA-locus	P[2] = 0.873800<br>P[3] = 0.126200	P[2] = 0.886560<br>P[3] = 0.113440	P[2] = 0.901820<br>P[3] = 0.098180	P[2] = 0.883440<br>P[3] = 0.116560	P[2] = 0.892350<br>P[3] = 0.107650	P[2] = 0.909660<br>P[3] = 0.090340
	nDNA-1000 loci	P[2] = 1.000000	P[2] = 1.000000	P[2] = 1.000000	P[2] = 1.000000	P[2] = 1.000000	P[2] = 1.000000
**A10**	mtDNA-locus	P[2] = 1.000000	P[2] = 1.000000	P[2]= 0.99999<br>P[3]= 0.00001	P[2] = 1.000000	P[2] = 1.000000	P[2] = 1.000000
	nDNA-1000 loci	P[2] = 1.000000	P[2] = 1.000000	P[2] = 1.000000	P[2] = 1.000000	P[2] = 1.000000	P[2] = 1.000000
**A11 on five sets of nDNA 20 loci**	nDNA-20loci set1	P[2] = 0.893825<br>P[3] = 0.106175	P[2] = 0.976994<br>P[3] = 0.023006	P[2] = 0.922345<br>P[3] = 0.077655	P[2] = 0.905784<br>P[3] = 0.094216	P[2] = 0.88896<br>P[3] = 0.11104	P[2] = 0.887968<br>P[3] = 0.112032
	nDNA-20loci set2	P[2] = 0.95253<br>P[3] = 0.04747	P[2] = 0.97203<br>P[3] = 0.02797	P[2] = 0.91916<br>P[3] = 0.08084	P[2] = 0.999608<br>P[3] = 0.000392	P[2] = 0.93108<br>P[3] = 0.06892	P[2] = 0.944306<br>P[3] = 0.055693
	nDNA-20loci set3	P[2] = 0.939535<br>P[3] =0.060465	P[2] = 0.915516<br>P[3] = 0.084483	P[2] = 0.83647<br>P[3] = 0.16353	P[2] = 0.919236<br>P[3] = 0.080763	P[2] = 0.86528<br>P[3] = 0.13472	P[2] = 0.989486<br>P[3] = 0.010513
	nDNA-20loci set4	P[2] = 1.000000	P[2] = 0.955943<br>P[3] =0.044057	P[2] = 0.999750<br>P[3] =0.000250	P[2] = 0.974528<br>P[3] =0.025472	P[2] = 1.000000	P[2] = 1.000000
	nDNA-20loci set5	P[2] = 0.892005<br>P[3] = 0.107995	P[2] = 0.871017<br>P[3] = 0.128983	P[2] = 0.897013<br>P[3] = 0.102988	P[2] = 0.958009<br>P[3] = 0.041991	P[2] = 0.891627<br>P[3] = 0.108373	P[2] = 0.830493<br>P[3] = 0.169507

Table S5. *A priori* list of 34 candidate Gene Symbols and Gene Names identified in the *C. tularosa* genome (GeneID). Gene orthologs are listed in the Gene Identifier column.

GeneID	Gene Symbol	Gene Name	Gene Identifier
119779682	aqp10b	aquaporin 10b	100034395
119785725	aqp11	aquaporin 11	282679
119772254	aqp12	aquaporin 12	436844
119773043	aqp3a	aquaporin 3a	406777
119795648	aqp4	aquaporin 4	361
119778548	aqp7	aquaporin 7	334529
119776015	aqp8a.2	aquaporin 8a, tandem duplicate 2	563130
119795465	aqp9b	aquaporin 9b	366
119778619	ca10a	carbonic anhydrase Xa	56934
119772393	ca12	carbonic anhydrase XII	771
119782954	ca14	carbonic anhydrase XIV	23632
119796736	ca15b	carbonic anhydrase XVb	791844
119775642	ca4a	carbonic anhydrase IV a	555196
119795333	ca4b	carbonic anhydrase IV b	553246
119792697	ca4c	carbonic anhydrase IV c	407683
119787243	ca5a	carbonic anhydrase Va	569989
119797057	ca6	carbonic anhydrase VI	100006448
119782661	ca7	carbonic anhydrase VII	766
119774183	ca8	carbonic anhydrase VIII	767
119789020	car15	carbonic anhydrase 15	568143
119780462	cftr	CF transmembrane conductance regulator	1080
119785913	nkain1	sodium/potassium transporting ATPase interacting 1	79570
119788681	nkain2	sodium/potassium transporting ATPase interacting 2	154215
119774097	nkain4	sodium/potassium transporting ATPase interacting 4	100002876
119797096	pgd	phosphogluconate dehydrogenase	5226
119796209	slc4a11	solute carrier family 4 member 11	83959
119785288	slc4a1a	solute carrier family 4 member 1a (Diego blood group)	84703
119792278	slc4a1b	solute carrier family 4 member 1b (Diego blood group)	561787

119776807	slc4a2a	solute carrier family 4 member 2a	566075
119791679	slc4a2b	solute carrier family 4 member 2b	570716
119775131	slc4a3	solute carrier family 4 member 3	100333073
119781488	slc4a5a	solute carrier family 4 member 5a	559268
119795963	slc4a5b	solute carrier family 4 member 5b	100006379
119785405	slc4a8	solute carrier family 4 member 8	557494

Table S6. Estimates of global per-site divergence (D_{xy}) values between *Cyprinodon tularosa* population pairs used in this study and between two other pupfish species (*C. diabolis* vs *C. nevadensis*) from a previously published study (21). The Malpais Spring population shows similar levels of divergence with Salt Creek/Lost River compared to a species level comparison (*C. diabolis* and *C. nevadensis)*.

**Population/Species comparison**	**D_{xy}**
Salt Creek-Lost River	7.0×10^{-4}
Salt Creek-Malpais Spring	1.30×10^{-3}
Lost River-Malpais Spring	1.27×10^{-3}
C. diabolis vs C. nevadensis	$1.03 \text{ x } 10^{-3}$
